# Efficacy and safety of targeted therapeutics for patients with radioiodine-refractory differentiated thyroid cancer: Systematic review and network meta-analysis

**DOI:** 10.3389/fphar.2022.933648

**Published:** 2022-08-26

**Authors:** Xiaoyu Ji, Weili Liang, Guixu Lv, Changyuan Ding, Hong Lai, Luchuan Li, Qingdong Zeng, Bin Lv, Lei Sheng

**Affiliations:** ^1^ Department of Thyroid Surgery, General Surgery, Qilu Hospital of Shandong University, Jinan, China; ^2^ Department of Endocrinology, Qilu Hospital of Shandong University, Jinan, China

**Keywords:** radioiodine-refractory, differentiated thyroid cancer, targeted therapy, lenvatinib (LEN), sorafenib, apatinib, cabozantinib, anlotinib

## Abstract

**Background:** Multiple targeted therapeutics are available for radioiodine-refractory differentiated thyroid cancer (RAIR-DTC), but it remains unclear which treatment is optimal to achieve long-term survival.

**Methods:** A systematic search of the PubMed, Embase, and ClinicalTrials.gov databases was conducted to identify eligible randomized controlled trials (RCTs) comparing the efficacy and safety of targeted treatments for patients with RAIR-DTC from inception to April, 2022. Data were extracted by following the recommendations of the Preferred Reporting Items for Systematic Review and Meta-analysis guidelines. We calculated the odds ratio (OR) or hazard ratio (HR), its corresponding 95% credible intervals (CrI), and the surface under the cumulative ranking curve (SUCRA) to indicate ranking probability using Bayesian network meta-analyses. The primary outcome was progression-free survival (PFS). The secondary outcomes were overall survival (OS), objective response rate (ORR), disease control rate (DCR), and grade 3 or higher adverse events.

**Results:** A total of 12 eligible RCTs involved 1,959 patients and 13 treatments: apatinib, cabozantinib, anlotinib, nintedanib, lenvatinib, lenvatinib with low dose (LD), sorafenib, sorafenib plus everolimus, donafenib (200 mg), donafenib (300 mg), pazopanib (continuous), pazopanib (intermittent), and vandetanib. Pooled analyses indicated that targeted therapeutics significantly prolonged PFS and OS in patients with RAIR-DTC (0.31, 0.21–0.41; 0.69, 0.53–0.85, respectively) compared with placebo. Network meta-analyses indicated that lenvatinib showed the most favorable PFS, with significant differences versus sorafenib (0.33, 0.23-0.48), vandetanib (0.31, 0.20–0.49), nintedanib (0.30, 0.15–0.60), and placebo (0.19, 0.15–0.25), while apatinib was most likely to be ranked first for prolonging OS with a SUCRA of 0.90. Lenvatinib showed the highest ORR (66%, 61%–70%), followed by anlotinib (59%, 48%–70%) and apatinib (54%, 40%–69%). Lenvatinib caused the most adverse events of grade 3 or higher, followed by lenvatinib (LD) and apatinib. Different toxicity profiles of individual treatment were also revealed.

**Conclusion:** This network meta-analysis suggests that lenvatinib and apatinib were associated with the best progression-free survival and overall survival benefits, respectively, for patients with RAIR-DTC, compared with other targeted therapeutics. Patients who received lenvatinib or apatinib also had more grade 3 or higher adverse events.

**Systematic Review Registration**: [https://www.crd.york.ac.uk/PROSPERO/display_record.php?RecordID=302249], identifier [CRD42022302249].

## Introduction

Thyroid cancer is the most frequent endocrine malignancy, and its incidence has gradually increased in the past decade (Siegel et al., 2018). Differentiated thyroid cancer (DTC) accounts for over 90% of all thyroid cancers and can be subdivided into follicular, papillary, Hürthle cell, and poorly differentiated thyroid cancers (Schlumberger and Leboulleux, 2021). The overall prognosis of thyroid cancer is usually favorable, with a 10-year survival rate up to 97% (Ito et al., 2018). Majority of patients with DTC can be cured by thyroidectomy and thyroid-stimulating hormone (TSH) suppression therapy, with or without radioactive iodine (RAI) therapy. However, about 30% of DTCs with locally advanced or distant diseases eventually become refractory to RAI treatment, which results in the 10-year survival rate less than 20% (Karapanou et al., 2022).

RAIR-DTC has limited therapeutic options and is insensitive to conventional cytotoxic drugs and systemic chemotherapy (Droz et al., 1990). But in recent years, with the in-depth study of the molecular mechanism and actionable gene mutations related to DTC, multiple therapeutic targets have been identified, opening a new era of targeted therapy for patients with RAIR-DTC. Lenvatinib and sorafenib, multiple kinase inhibitors (MKIs), were found to significantly prolong progression-free survival (PFS) and were recommended for patients with RAIR-DTC in 2015 by American Thyroid Association (ATA) thyroid cancer guidelines (Brose et al., 2014; Schlumberger et al., 2015; Haugen et al., 2016). Recently, several novel MKIs, such as apatinib, anlotinib, and cabozantinib, have been developed and assessed in the management of RAIR-DTC as compared to placebo in a series of phase II/III randomized controlled trials (RCTs) (Chi et al., 2020; Brose et al., 2021; Lin et al., 2022). Nevertheless, the relative effects of any of these MKIs compared with another in patients with RAIR-DTC remain elusive due to lack of direct evidence from head-to-head RCTs.

To investigate the relative efficacy and safety of all the targeted therapeutics, we conducted this network meta-analysis (NMA) to compare data from both direct and indirect comparisons. In addition, the Bayesian approach enables us to estimate the ranking probability of each treatment, providing guidance for clinicians to select the optimal treatment option for patients with RAIR-DTC.

## Methods

This NMA was performed in accordance with the PRISMA extension statement for NMA (Supplementary Table S1) and registered with PROSPERO (CRD42022302249).

### Data sources and searches

PubMed, Embase, and ClinicalTrials.gov databases were searched to find relevant articles up to April 2022. Abstracts on thyroid cancer from several important international conferences (the American Society of Clinical Oncology and European Society of Medical Oncology) from 2015 to 2021 were inspected to identify potentially relevant studies. Articles in English were included. The detailed search strategy is shown in Supplementary Material.

### Eligibility criteria

We included published phase II or III RCTs, including at least one arm with targeted therapy that met the following inclusion criteria: 1) pathologically confirmed differentiated thyroid cancer as papillary, follicular, Hürthle cell thyroid cancer, or poorly differentiated thyroid cancer. 2) Evidence of radioactive iodine^131^ refractory disease or patients with locally advanced or metastatic disease. 3) 16 years of age or older. We excluded trials with radiotherapy or chemotherapy only but without targeted therapy and phase II trials with single-arm treatment without comparison. We also excluded trials with histological subtypes of thyroid carcinoma other than the differentiated type and trials that studied specific populations, such as limited regions or age or lack of necessary outcomes.

### Data extraction

Main data on qualified trials such as study ID, first author, region, study phase, sample size, median age, sex of patients, patient proportion receiving prior targeted therapy, treatments, and reported outcomes were extracted into a spreadsheet for further analysis. All investigators independently extracted data parameters using a unified data extraction form.

The primary outcome was PFS (defined as the time from randomization to disease progression or death from any cause). Secondary outcomes were overall survival (OS) (time from randomization to death from any cause), objective response rate (ORR) (proportion of patients with a confirmed complete response (CR) or partial response (PR) from randomization to disease progression or death from any cause), disease control rate (DCR), and grade 3 or higher adverse events (⩾3 AEs). All available direct and indirect evidence was synthesized to compare different treatments in terms of efficacy and safety, reported as the hazard ratio (HR)/odds ratio (OR) and corresponding 95% credible intervals (CrI) for OS, PFS, ORR, DCR, and ⩾3 AEs.

### Risk of bias assessment

Two investigators (WLL and XYJ) independently assessed the risk of bias of individual studies. Any disagreement was discussed and resolved by BL and LS to reach a consensus. The bias risk of included trials was assessed using the Cochrane risk of bias tool, consisting of random sequence generation, allocation concealment, blinding of participants and personnel, blinding of outcome assessment, incomplete outcome data, selective outcome reporting, and other sources of bias.

### Data synthesis and analysis

Network plots were generated by Stata 15.0, choosing which treatments were compared directly or indirectly for different outcomes. NMAs were performed in a Bayesian framework using a Markov Chain Monte Carlo simulation technique within the GEMTC package in the R-Statistics and the J.A.G.S. program, as previously described (Sheng et al., 2021). Fixed-effect models were used since in most cases, the treatment of interest was evaluated in a single trial (Dafni et al., 2019). We used non-informative uniform and normal prior distributions to fit the model, with four different sets of initial values. For each outcome, 150, 000 sample iterations were generated with 100, 000 burn-ins and a thinning interval of 1, except for DCR and ⩾3 AEs, for which we increased the thinning interval to 10 to minimize auto-correlation. We tested the adequacy of convergence using trace plots and estimated the values of the Brooks–Gelman–Rubin statistic (Supplementary Figure S1). Once the convergence was established, the posterior distributions for the model parameters were obtained. Pairwise meta-analyses were further performed with the frequentist method for head-to-head trials based on two comparisons. Heterogeneity was assessed between studies using the Q test and *I* ^2^ statistic. The estimated *I* ^2^ values under 25%, between 25% and 50%, or over 50% indicated low, moderate, or high heterogeneity, respectively (Higgins et al., 2003). Since the zero-count event is frequent in comparison of ORR, we performed meta-analysis by pooling the risk ratio of each treatment instead of conducting network meta-analysis. We reconstructed a novel Kaplan–Meier survival curve under the same treatment and calculated median PFS and OS using the graphic reconstructive algorithm with packages of IPD from KM, survival, tableone, and ggplot2 (Wei and Royston, 2017; Villanueva and Chen, 2019; Liu et al., 2021).

The probability of being at each rank was estimated for all treatments. A treatment hierarchy was determined using the probability of being the best treatment by using the surface under the cumulative ranking curve (SUCRA; score of 0–1 and 1 is the best) (Zhao et al., 2019).

### Sensitivity analysis

In addition to the principal analysis, a sensitivity analysis was conducted to test the robustness and reliability of results by excluding trials with 100% of patients receiving prior targeted therapy (Schlumberger et al., 2018; Brose et al., 2021).

## Results

### Literature search and study characteristics

**FIGURE 1:**
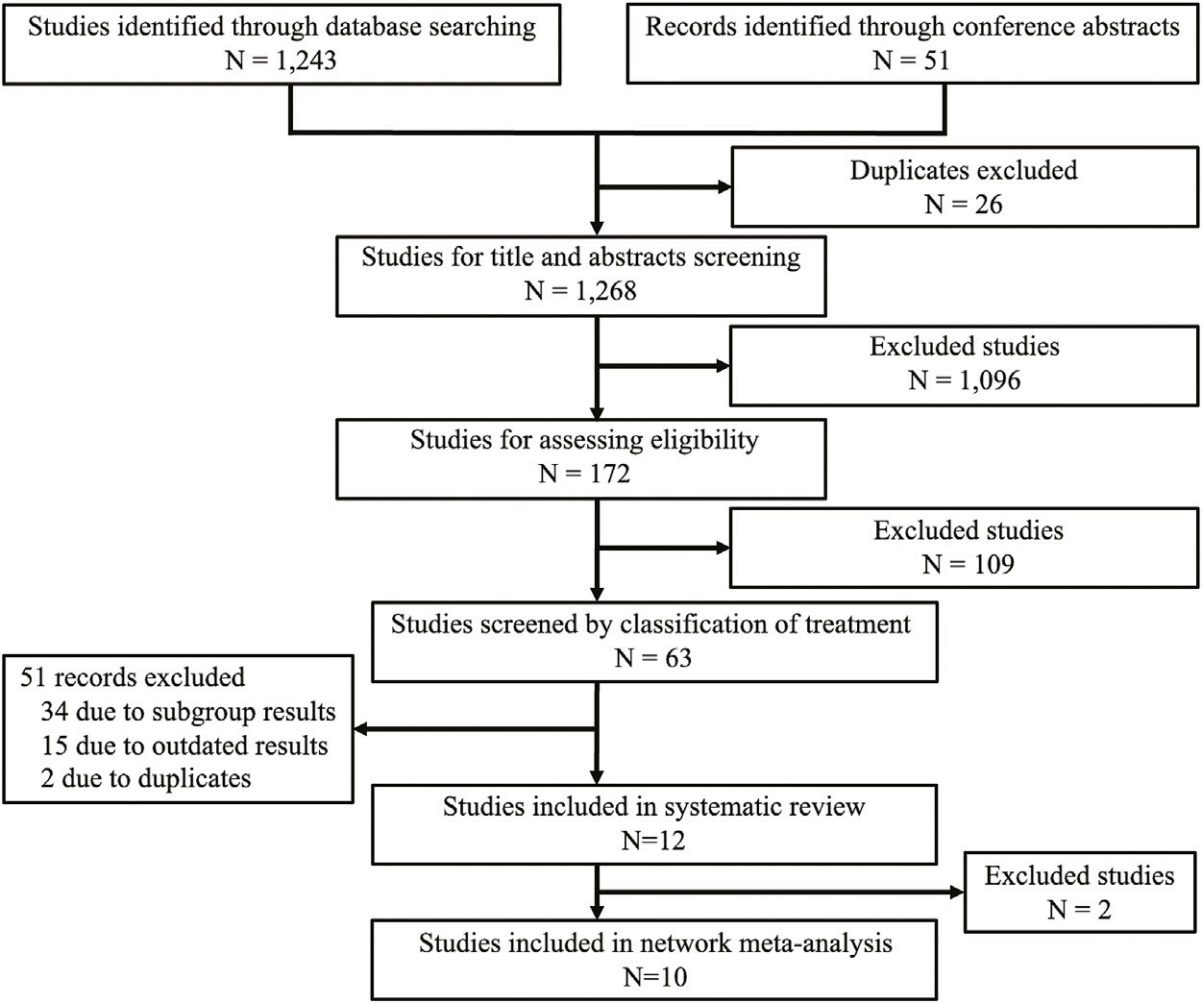
Study selection flowchart.

**FIGURE 2:**
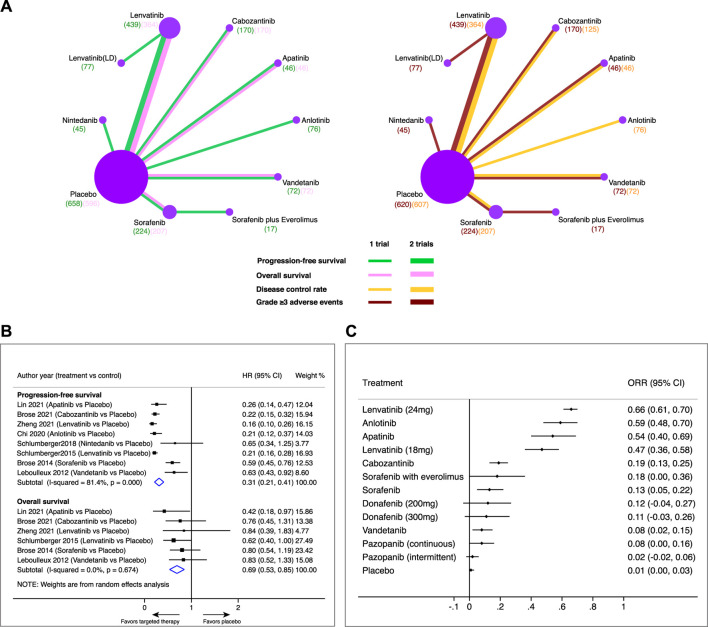
Pairwise meta-analysis of a comparison of targeted therapy versus placebo on major outcomes in patients with radioiodine-refractory differentiated thyroid cancer. **(A)** Network diagrams of comparing progression-free survival (PFS), overall survival (OS), disease control rate (DCR), and grade 3 or higher adverse events (⩾3 AEs). Each circular node represents a type of treatment. Each line represents a type of head-to-head comparison. Node size and line thickness are weighted according to the number of studies evaluating each treatment and direct comparison, respectively. The total number of patients receiving a treatment is shown in brackets. **(B)** Pooled hazard ratio (HR) of PFS and OS in comparison of targeted therapy versus placebo. **(C)** Objective response rate (ORR) and its 95% confidence interval (CI) for each treatment.

Twelve trials were included, involving 1,959 patients and a total of 13 active treatment regimens (Table 1) (Leboulleux et al., 2012; Brose et al., 2014; Schlumberger et al., 2015; Schlumberger et al., 2018; Brose et al., 2020; Chi et al., 2020; Brose et al., 2021; Capdevila et al., 2021; de la Fouchardière et al., 2021; Lin et al., 2021; Sherman et al., 2021; Zheng et al., 2021; Lin et al., 2022). The study selection diagram is shown in Figure 1. They are targeted therapy drugs which include apatinib, cabozantinib, anlotinib, nintedanib, lenvatinib, low-dose lenvatinib (LD), sorafenib, sorafenib plus everolimus, donafenib (200 mg), donafenib (300 mg), pazopanib (continuous), pazopanib (intermittent), and vandetanib. All included studies were RCTs, of which REALITY (Lin et al., 2022), COSMIC-311 (Brose et al., 2021; Capdevila et al., 2021), Zheng 2021 (Zheng et al., 2021), SELECT (Schlumberger et al., 2015), and DECISION (Brose et al., 2014) were phase III clinical trials. Total two RCTs were excluded for network meta-analysis as one study evaluated the two dosages of donafenib, and another study compared continuous versus intermittent administration of pazopanib (de la Fouchardière et al., 2021; Lin et al., 2021). The networks involving 10 different treatments are shown in Figure 2A. The detailed risk of bias assessment of each trial is summarized in Supplementary Figure S2.

**TABLE 1:** Baseline characteristics of trials included in the network meta-analysis of patients with radioiodine-refractory differentiated thyroid cancer.

Study	Region	Phase	Sample size	Median age	Male/female	Prior targeted therapy, n (%)	Intervention arm	Control arm	Reported outcomes
Lin et al. (2021)	China	III	46/46	56/59.5	36/56	8 (8.7)	Apatinib	Placebo	PFS, OS, ORR, DCR, and ⩾3 AEs
Brose et al. (2021)	Europe, Asia, North America, etc.	III	170/88	65/66	NA	258 (100)	Cabozantinib	Placebo	PFS, OS, ORR, DCR, and ⩾3 AEs
Zheng et al. (2021)	China	III	103/48	61/60	78/73	38 (25.2)	Lenvatinib	Placebo	PFS, OS, ORR, DCR, and ⩾3 AEs
Sherman et al. (2021)	United States	II	17/17	66.5	NA	NA	Sorafenib and everolimus	Sorafenib	PFS, ORR, and ⩾3 AEs
Fouchardière et al. (2021)	France	II	50/50	67.0/65.5	53/47	27 (27)	Pazopanib (intermittent)	Pazopanib (continuous)	PFS, ORR, and ⩾3 AEs
Lin et al. (2020)	China	II	17/18	56.47/54.28	13/22	0 (0)	Donafenib (200 mg)	Donafenib (300 mg)	PFS, ORR, and DCR, ⩾3 AEs
Chi et al. (2020)	China	II	76/37	NA	NA	0 (0)	Anlotinib	Placebo	PFS, ORR, and DCR
Brose et al. (2020)	North America, Europe, Asia, etc.	II	75/77	64.3/64.4	78/74	NA	Lenvatinib (24 mg)	Lenvatinib (18 mg)	PFS, ORR, and ⩾3 AEs
Schlumberger et al. (2018)	France	II	45/25	65.8	NA	70 (100)	Nintedanib	Placebo	PFS and ⩾3 AEs
Schlumberger (2015)	Europe, North America, Asia, etc.	III	261/131	64/61	200/192	93 (23.7)	Lenvatinib	Placebo	PFS, OS, ORR, DCR, and ⩾3 AEs
Brose et al. (2014)	Europe, North America, and Asia	III	207/210	63/63	199/218	13 (3.1)^a^	Sorafenib	Placebo	PFS, OS, ORR, DCR, and ⩾3 AEs
Leboulleux et al. (2012)	Europe	II	72/73	63/64	78/67	6 (4.1)	Vandetanib	Placebo	PFS, OS, ORR, DCR, and ⩾3 AEs

a Any prior systemic anticancer therapy. PFS, progression-free survival. OS, overall survival. ORR, objective response rate. DCR, disease control rate. ⩾3 AEs, grade 3 or higher adverse events.

### Efficacy

A total of eight trials involving 1,347 patients were available for assessing PFS in the comparison of targeted therapeutics with a placebo (Figure 2B). Pooled analysis indicated that targeted therapeutics significantly prolonged PFS compared with placebo (HR 0.31, 95% CI 0.21–0.41). Likewise, a total of six trials involving 1,455 patients were available for assessing OS in comparison of targeted therapeutics with placebo (Figure 2B). Pooled analysis showed that targeted therapeutics significantly improved OS compared with placebo (HR 0.69, 95% CI 0.53–0.85). Similar results were obtained when fixed models were used for PFS (HR 0.24, 95% CI 0.20–0.27) and OS (HR 0.69, 95% CI 0.53–0.85), respectively (Supplementary Figure S3), indicating the robustness of the pooled results. Lenvatinib showed the highest ORR (66%, 95% CI 61%–70%), followed by anlotinib (59%, 95% CI 48%–70%), apatinib (54%, 95% CI 40%–69%), and lenvatinib (LD) (47%, 95% CI 36%–58%), while the ORRs of the other treatments were less than 20% (Figure 2C).

**FIGURE 3:**
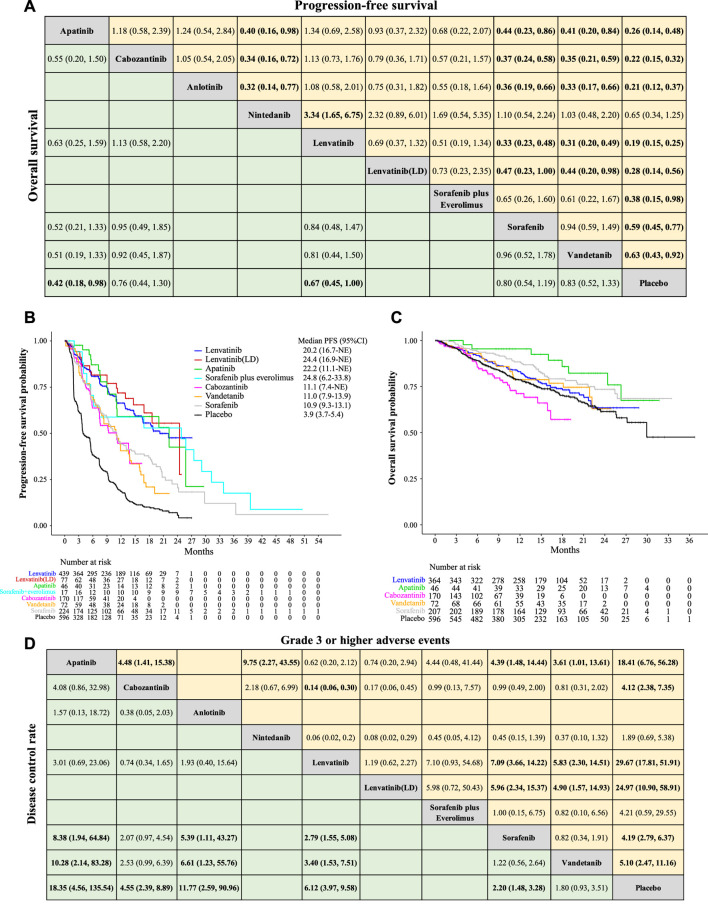
Network meta-analysis of a comparison of different treatments on PFS, OS, DCR, and ⩾3 AEs in patients with radioiodine-refractory differentiated thyroid cancer. **(A)** Pooled estimates of the network meta-analysis of progression-free survival (PFS) and overall survival (OS). Data on each cell is a hazard ratio (HR) (95% CrIs) for the comparison of row-defining treatment versus column-defining treatment. The HR less than 1 favors upper-row treatment. Significant results are highlighted in bold. **(B)** Reconstruction of the Kaplan–Meier curve of PFS for each treatment with median PFS. **(C)** Reconstruction of the Kaplan–Meier curve of OS for each treatment. **(D)** Pooled estimates of the network meta-analysis of the disease control rate (DCR) and grade 3 or higher adverse events (⩾3 AEs). Data on each cell is an odds ratio (OR) (95% CrIs) for the comparison of row-defining treatment versus column-defining treatment. The OR greater than 1 favors upper-row treatment. Significant results are highlighted in bold.

In terms of PFS (Figures 3A,B), median PFS derived from the Kaplan–Meier survival curve in each study was consistent with the originally reported PFS (Supplementary Table S2), indicating the reliability of the methodology. The reconstructed median PFS of each treatment is displayed in Figure 3B. Lenvatinib showed the most favorable PFS (median PFS, 20.2 months, and 95% CI 16.7-NE), with significant differences versus sorafenib (HR 0.33, 95% CrI 0.23–0.48), vandetanib (HR 0.31, 95% CrI 0.20–0.49), nintedanib (HR 0.30, 95% CrI 0.15–0.60), and placebo (HR 0.19, 95% CrI 0.15–0.25). Anlotinib, cabozantinib, and apatinib were shown to be consistent with lenvatinib in providing the PFS benefits (HR 1.34, 95% CrI 0.69–2.58; HR 1.13, 95% CrI 0.73–1.76; HR 1.08, 95% CrI 0.58–2.01, respectively).

In terms of OS (Figures 3A,C), apatinib and lenvatinib were consistent (HR 0.63, 95% CrI 0.25–1.59) in providing the best OS benefit. Significant differences were also observed when compared with placebo (HR 0.42, 95% CrI 0.18–0.98 for apatinib; HR 0.67, 95% CrI 0.45–1.00 for lenvatinib). A similar efficacy was found between cabozantinib, sorafenib, and vandetanib since the hazard ratios were close to 1. Within the duration of the follow-up, median OS was not reached for all the active treatments.

In terms of DCR (Figure 3D), no significant difference was observed among lenvatinib, anlotinib, and apatinib; however, they consistently produced significant DCR benefits over sorafenib, vandetanib, and placebo. Furthermore, apatinib was likely to be the best treatment in achieving disease control, followed by anlotinib and lenvatinib.

### Safety—adverse events (AEs)

All included studies reported significantly higher rates of grade 3 or higher adverse events (≥3 AEs) after using targeted therapeutics compared with placebo. Lenvatinib had the highest rate of ≥3 AEs, followed by lenvatinib (LD) and apatinib (Figure 3D). Commonly reported ≥3 AEs for lenvatinib are hypertension (47.5%), proteinuria (13.7%), diarrhea (7.7%), and fatigue (6.6%) (Supplementary Table S3). Different toxicity profiles of individual treatments are also available in Supplementary Table S3.

### Rank probabilities

**FIGURE 4:**
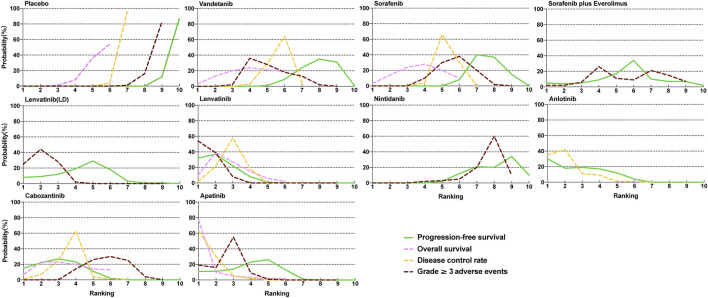
Profiles indicate the probability of each comparable treatment being ranked from first to last on overall survival, progression-free survival, disease control rate, and grade 3 or higher adverse events.

The Bayesian ranking profiles of comparable treatments are summarized in Figure 4 and Supplementary Figure S4. For patients with RAIR-DTC, lenvatinib was most likely to be ranked first for PFS (cumulative probability of 88%), apatinib for both OS (90%) and DCR (92%), and lenvatinib for ≥3 AEs (93%).

### Sensitive analysis

To test the reliability and robustness of the results, sensitive analysis of network meta-analysis was performed by excluding two trials including patients with 100% of previously treated patients with targeted therapy (Schlumberger et al., 2018; Brose et al., 2021). The results of the sensitive analysis remain the same as the primary analysis (Supplementary Figure S5).

## Discussion

In this systematic review and network meta-analysis, we comprehensively compared the efficacy and safety of multiple targeted therapeutics for patients with RAIR-DTC. The pooled results suggested that targeted therapeutics significantly prolonged PFS and OS in patients with RAIR-DTC compared with placebo. Among all active treatments, lenvatinib and apatinib were associated with the best PFS and OS improvement, respectively, but with more grade 3 or higher adverse events.

Our network meta-analysis indicated that lenvatinib was associated with the best PFS improvement, followed by anlotinib, cabozantinib, and apatinib. In addition, our analysis also suggested that lenvatinib was significantly better in improving PFS than sorafenib (HR = 0.33, 95% CrI 0.23–0.48), which is consistent with the previous result from the indirect comparison of SELECT and DECISION trials (HR = 0.36, 95% CI 0.22–0.57) (Kawalec et al., 2016). In accordance with their role in PFS improvement, lenvatinib, anlotinib, and apatinib showed a high and comparable ORR, while the ORR is less than 20% in the remaining therapeutic options. The majority of RCTs included patients with previously untreated or limited cases (less than 30%) of previously treated with tyrosine kinase inhibitors, except for COSMIC-311 and Schlumberger 2018 with 100% of prior targeted therapy. It should be noted that the study population of COSMIC-311 was pretreated with sorafenib or lenvatinib, resulting in shorter median PFS and relatively lower ORR than the first-line therapeutic options such as lenvatinib in other studies. A previous phase 2 study was conducted to assess cabozantinib as the first-line treatment in RAIR-DTC (Brose et al., 2018). A total of 54% (19/35) of patients achieved objective response with a median duration of the response of 40 weeks, which is comparable with the other first-line therapeutic options, such as lenvatinib (66%), anlotinib (59%), and apatinib (54%). Overall survival outcomes of most trials included were immature, and interim analysis found no statistically significant differences among all data available on active treatments. However, Bayesian ranking probability analysis indicated that apatinib was most likely to be ranked first in improving OS, followed by lenvatinib, with both being significantly superior to placebo.

Multikinase inhibitors, such as lenvatinib and sorafenib, are currently the standard-of-care for the RAIR-DTC. Although they have shown favorable clinical activity, they are associated with significant adverse events, resulting in frequent dose reduction and discontinuation (Al-Jundi et al., 2020). According to our network meta-analysis, lenvatinib caused the highest incidence of grade 3 or higher adverse events, followed by apatinib, although lenvatinib achieved the best PFS and apatinib showed the best OS. The incidences of grade 3 or adverse events in the lenvatinib group and in the apatinib group were 78.6% and 73.9%, leading to 13% and 6.5% of discontinuation of treatment, respectively. The most common grade 3 or higher treatment-related adverse event for both lenvatinib and apatinib was hypertension (47.5% *vs*. 34.8%). In addition, preliminary data suggested that anlotinib has also achieved encouraging median PFS and ORR in patients with RAIR-DTC before releasing its final safety profile. However, the safety profile of anlotinib was available in a phase IIB study involving 62 patients with locally advanced or metastatic medullary thyroid cancer (Li et al., 2021). The incidence of grade 3 or higher treatment-related adverse events was 58.1% in the anlotinib group, including hand-foot syndrome (12.9%), hypertension (11.3%), and elevated lipase (9.7%). Discontinuation of anlotinib owing to adverse events occurred in 16.1% (10/62) of patients. Sorafenib, cabozantinib, and vandetanib showed similar and relatively lower incidence of grade 3 or higher treatment-related adverse events, but their efficacy was also compromised. Frequent treatment-related adverse events of the aforementioned multikinase inhibitors are primarily due to their broad activity against many kinases. Thus, the development of highly selective inhibitors targeting a specific receptor or molecular pathway of thyroid cancer is an unmet need and may be a plausible way to ensure a durable response without increasing side effects.

Recently, several highly selective inhibitors have been developed and assessed in thyroid cancer harboring actionable genetic alterations. *BRAF* mutations are the most common genetic alterations in thyroid cancer (59.7%), while *RET* fusions are present in about 10%–20% of differentiated thyroid cancer (Romei et al., 2008; Network, 2014; Pozdeyev et al., 2018). Vemurafenib, a selective BRAF inhibitor, showed an intermediate overall response in patients with RAIR-DTC harboring *BRAF* mutation in both treatment-naïve (38.5%, 10 of 26) patients and those previously treated with a multikinase inhibitor (27.3%, 6 of 22) (Brose et al., 2016). Another BRAF inhibitor dabrafenib showed similar response (29%, 4 of 14) in PTC patients with *BRAF* mutation (Falchook et al., 2015). Similar to melanoma, acquired activating *MEK1* mutation or overexpression of HER2/3 may confer resistance to the single BRAF inhibition (Wagle et al., 2011). Several trials are ongoing to investigate the combined efficacy of BRAF inhibitors (e.g., dabrafenib) and MEK inhibitors (e.g., trametinib), or HER3 inhibitors (e.g., lapatinib) for patients with *BRAF*-mutated RAIR-DTC (NCT01723202, NCT03244956, and NCT01947023). Moreover, two novel selective RET inhibitors, selpercatinib and pralsetinib, have been shown to have potent antitumor activity in thyroid cancer with *RET* alterations (Wirth et al., 2020; Subbiah et al., 2021). In 19 patients with previously treated *RET* fusion-positive thyroid cancer, selpercatinib showed a 79% objective response and 64% of 1-year PFS rate, with a low discontinuous rate (2%, 12 out of 531) due to adverse events (Wirth et al., 2020). Similarly, pralsetinib also showed an encouraging overall objective response rate (89%, 8 of 9) for patients with *RET* fusion-positive thyroid cancer. Although median PFS and OS were not reached, estimated 1-year PFS and OS rates were 81% and 91%, respectively. Of all 142 patients treated, 5 (4%) discontinued pralsetinib, owing to drug-related adverse events (Subbiah et al., 2021). In addition, as potent inhibitors of tropomyosin receptor kinase (TRK) A, B, and C, entrectinib and larotrectinib revealed 50% (2 of 4) and 100% (5 of 5) of overall objective response in thyroid cancer harboring *NTRK* gene fusion, respectively (Doebele et al., 2020). Therefore, molecular screening for patients with RAIR-DTC will be essential in identifying a subset of patients with genetic alterations who may benefit from highly selective inhibitors without compromising their safety profile.

The strength of this study is the inclusion of the most up-to-date RCTs with a low or medium risk of bias. Our comprehensive pairwise and network meta-analysis revealed that targeted therapeutics significantly prolonged the long-term survival in patients with RAIR-DTC and compared the relative efficacy and safety of each active treatment. Sensitivity analysis was performed to ensure the robustness and reliability of the results. However, this study also has several limitations. First, although all the studies in our analysis included patients with RAIR-DTC, some studies included a few patients with different driver-gene mutated RAIR-DTC or previously received radiation, chemotherapy, or other types of targeted therapeutics, which may have some impact on the outcome. Second, the OS data on some studies were immature and extracted or calculated from interim reports or the latest meeting abstracts. Third, some therapeutic regimes, such as apatinib, anlotinib, nintedanib, and vandetanib, were assessed in a single country with a limited number of patients included, which is a critical source of inter-trial heterogeneity. Further RCTs are warranted to assess their efficacy and safety in a broader population, and the optimal treatment regimen for RAIR-DTC may be evolving as more RCTs are released. Fourth, the main subjects included in this analysis were differentiated thyroid cancers, including papillary, follicular, Hürthle cell thyroid cancer, and poorly differentiated thyroid cancers. However, it remains unclear about the optimal therapeutic regimen for each histological subtype of DTC as subgroup analyses were not performed in most trials included.

## Conclusion

In conclusion, this study suggests that the targeted therapeutics showed a delightful efficacy for RAIR-DTC as compared with placebo. Lenvatinib was associated with the best PFS improvement, while apatinib showed the best OS, compared with the other active targeted therapeutics. Patients who received lenvatinib or apatinib also had more adverse events. More high-quality head-to-head RCTs are required to evaluate the efficacy and safety of targeted agents for patients with RAIR-DTC.

## Data Availability

The original contributions presented in the study are included in the article/Supplementary Material; further inquiries can be directed to the corresponding authors.

## References

[B1] Al-Jundi M. Thakur S. Gubbi S. Klubo-Gwiezdzinska J. (2020). Novel targeted therapies for metastatic thyroid cancer-A comprehensive review. Cancers (Basel) 12, 2104. 10.3390/cancers12082104 PMC7463725 32751138

[B2] Brose M. Panaseykin Y. Konda B. de la Fouchardiere C. Hughes B. Gianoukakis A. (2020). 426P A multicenter, randomized, double-blind, phase II study of lenvatinib (LEN) in patients (pts) with radioiodine-refractory differentiated thyroid cancer (RR-DTC) to evaluate the safety and efficacy of a daily oral starting dose of 18 mg vs 24 mg. Ann. Oncol. 31, S1409. 10.1016/j.annonc.2020.10.418

[B3] Brose M. S. Cabanillas M. E. Cohen E. E. Wirth L. J. Riehl T. Yue H. (2016). Vemurafenib in patients with BRAF(V600E)-positive metastatic or unresectable papillary thyroid cancer refractory to radioactive iodine: a non-randomised, multicentre, open-label, phase 2 trial. Lancet. Oncol. 17, 1272–1282. 10.1016/S1470-2045(16)30166-8 27460442 PMC5532535

[B4] Brose M. S. Nutting C. M. Jarzab B. Elisei R. Siena S. Bastholt L. (2014). Sorafenib in radioactive iodine-refractory, locally advanced or metastatic differentiated thyroid cancer: a randomised, double-blind, phase 3 trial. Lancet 384, 319–328. 10.1016/S0140-6736(14)60421-9 24768112 PMC4366116

[B5] Brose M. S. Robinson B. Sherman S. I. Krajewska J. Lin C-C. Vaisman F. (2021). Cabozantinib for radioiodine-refractory differentiated thyroid cancer (COSMIC-311): A randomised, double-blind, placebo-controlled, phase 3 trial. Lancet. Oncol. 22, 1126–1138. 10.1016/S1470-2045(21)00332-6 34237250

[B6] Brose M. S. Shenoy S. Bhat N. Harlacker A. K. Yurtal R. K. Posey Z. A. (2018). A phase II trial of cabozantinib (CABO) for the treatment of radioiodine (RAI)-refractory differentiated thyroid carcinoma (DTC) in the first-line setting. J. Clin. Oncol. 36, 6088. 10.1200/jco.2018.36.15_suppl.6088

[B7] Capdevila J. Robinson B. Sherman S. I. Jarząb B. Lin C. C. Vaisman F. (2021). LBA67 Cabozantinib versus placebo in patients with radioiodine-refractory differentiated thyroid cancer who have progressed after prior VEGFR-targeted therapy: Updated results from the phase III COSMIC-311 trial and prespecified subgroup analyses by prior therapy. Ann. Oncol. 32, S1343. 10.1016/j.annonc.2021.08.2148

[B8] Chi Y. Gao M. Zhang Y. Shi F. Cheng Y. Guo Z. (2020). 265O anlotinib in locally advanced or metastatic radioiodine-refractory differentiated thyroid carcinoma: A randomized, double-blind, multicenter phase II trial. Ann. Oncol. 31, S1347. 10.1016/j.annonc.2020.10.259 PMC10570678 37594724

[B9] Dafni U. Tsourti Z. Vervita K. Peters S. (2019). Immune checkpoint inhibitors, alone or in combination with chemotherapy, as first-line treatment for advanced non-small cell lung cancer. A systematic review and network meta-analysis. Lung cancer 134, 127–140. 10.1016/j.lungcan.2019.05.029 31319971

[B10] de la Fouchardière C. Godbert Y. Dalban C. Illouz F. Wassermann J. Do Cao C. (2021). Intermittent versus continuous administration of pazopanib in progressive radioiodine refractory thyroid carcinoma: Final results of the randomised, multicenter, open-label phase II trial PAZOTHYR. Eur. J. Cancer 157, 153–164. 10.1016/j.ejca.2021.07.029 34509954

[B11] Doebele R. C. Drilon A. Paz-Ares L. Siena S. Shaw A. T. Farago A. F. (2020). Entrectinib in patients with advanced or metastatic NTRK fusion-positive solid tumours: integrated analysis of three phase 1-2 trials. Lancet. Oncol. 21, 271–282. 10.1016/S1470-2045(19)30691-6 31838007 PMC7461630

[B12] Droz J. P. Schlumberger M. Rougier P. Ghosn M. Gardet P. Parmentier C. (1990). Chemotherapy in metastatic nonanaplastic thyroid cancer: experience at the institut gustave-roussy. Tumori 76, 480–483. 10.1177/030089169007600513 2256195

[B13] Falchook G. S. Millward M. Hong D. Naing A. Piha-Paul S. Waguespack S. G. (2015). BRAF inhibitor dabrafenib in patients with metastatic BRAF-mutant thyroid cancer. Thyroid 25, 71–77. 10.1089/thy.2014.0123 25285888 PMC4291160

[B14] Haugen B. R. Alexander E. K. Bible K. C. Doherty G. M. Mandel S. J. Nikiforov Y. E. (2016). 2015 American thyroid association management guidelines for adult patients with thyroid nodules and differentiated thyroid cancer: The American thyroid association guidelines task force on thyroid nodules and differentiated thyroid cancer. Thyroid 26, 1–133. 10.1089/thy.2015.0020 26462967 PMC4739132

[B15] Higgins J. P. Thompson S. G. Deeks J. J. Altman D. G. (2003). Measuring inconsistency in meta-analyses. BMJ 327, 557–560. 10.1136/bmj.327.7414.557 12958120 PMC192859

[B16] Ito Y. Miyauchi A. Kihara M. Fukushima M. Higashiyama T. Miya A. (2018). Overall survival of papillary thyroid carcinoma patients: A single-institution long-term follow-up of 5897 patients. World J. Surg. 42, 615–622. 10.1007/s00268-018-4479-z 29349484 PMC5801380

[B17] Karapanou O. Simeakis G. Vlassopoulou B. Alevizaki M. Saltiki K. (2022). Advanced RAI-refractory thyroid cancer: an update on treatment perspectives. Endocr. Relat. Cancer 29, R57–R66. 10.1530/ERC-22-0006 35266878

[B18] Kawalec P. Malinowska-Lipień I. Brzostek T. Kózka M. (2016). Lenvatinib for the treatment of radioiodine-refractory differentiated thyroid carcinoma: a systematic review and indirect comparison with sorafenib. Expert Rev. Anticancer Ther. 16, 1303–1309. 10.1080/14737140.2016.1247697 27734713

[B19] Leboulleux S. Bastholt L. Krause T. de la Fouchardiere C. Tennvall J. Awada A. (2012). Vandetanib in locally advanced or metastatic differentiated thyroid cancer: a randomised, double-blind, phase 2 trial. Lancet. Oncol. 13, 897–905. 10.1016/S1470-2045(12)70335-2 22898678

[B20] Li D. Chi Y. Chen X. Ge M. Zhang Y. Guo Z. (2021). Anlotinib in locally advanced or metastatic medullary thyroid carcinoma: A randomized, double-blind phase IIB trial. Clin. Cancer Res. 27, 3567–3575. 10.1158/1078-0432.CCR-20-2950 33832949

[B21] Lin Y. Qin S. Li Z. Yang H. Fu W. Li S. (2022). Apatinib vs placebo in patients with locally advanced or metastatic, radioactive iodine–refractory differentiated thyroid cancer: The REALITY randomized clinical trial. JAMA Oncol. 8, 242–250. 10.1001/jamaoncol.2021.6268 34913959 PMC8678901

[B22] Lin Y. S. Yang H. Ding Y. Cheng Y. Z. Shi F. Tan J. (2021). Donafenib in progressive locally advanced or metastatic radioactive iodine-refractory differentiated thyroid cancer: results of a randomized, multicenter phase II trial. Thyroid 31, 607–615. 10.1089/thy.2020.0235 32907500

[B23] Liu N. Zhou Y. Lee J. J. (2021). IPDfromKM: reconstruct individual patient data from published kaplan-meier survival curves. BMC Med. Res. Methodol. 21, 111. 10.1186/s12874-021-01308-8 34074267 PMC8168323

[B24] Network C. G. A. R. (2014). Integrated genomic characterization of papillary thyroid carcinoma. Cell 159, 676–690. 10.1016/j.cell.2014.09.050 25417114 PMC4243044

[B25] Pozdeyev N. Gay L. M. Sokol E. S. Hartmaier R. Deaver K. E. Davis S. (2018). Genetic analysis of 779 advanced differentiated and anaplastic thyroid cancers. Clin. Cancer Res. 24, 3059–3068. 10.1158/1078-0432.CCR-18-0373 29615459 PMC6030480

[B26] Romei C. Ciampi R. Faviana P. Agate L. Molinaro E. Bottici V. (2008). BRAFV600E mutation, but not RET/PTC rearrangements, is correlated with a lower expression of both thyroperoxidase and sodium iodide symporter genes in papillary thyroid cancer. Endocr. Relat. Cancer 15, 511–520. 10.1677/ERC-07-0130 18509003

[B27] Schlumberger M. Leboulleux S. (2021). Current practice in patients with differentiated thyroid cancer. Nat. Rev. Endocrinol. 17, 176–188. 10.1038/s41574-020-00448-z 33339988

[B28] Schlumberger M. Newbold K. Hasan B. Marreaud S. Assele S. Licitra L. F. (2018). A randomized doubled blind phase II study exploring the safety and efficacy of nintedanib (BIBF1120) as second line therapy for patients (pts) with differentiated thyroid carcinoma (DTC) progressing after first line therapy: EORTC 1209. J. Clin. Oncol. 36, 6021. 10.1200/jco.2018.36.15_suppl.6021

[B29] Schlumberger M. Tahara M. Wirth L. J. Robinson B. Brose M. S. Elisei R. (2015). Lenvatinib versus placebo in radioiodine-refractory thyroid cancer. N. Engl. J. Med. 372, 621–630. 10.1056/NEJMoa1406470 25671254

[B30] Sheng L. Gao J. Xu Q. Zhang X. Huang M. Dai X. (2021). Selection of optimal first-line immuno-related therapy based on specific pathological characteristics for patients with advanced driver-gene wild-type non-small cell lung cancer: a systematic review and network meta-analysis. Ther. Adv. Med. Oncol. 13, 17588359211018537. 10.1177/17588359211018537 34104227 PMC8165528

[B31] Sherman E. J. Foster N. R. Su Y. B. Shergill A. Ho A. L. Konda B. (2021). Randomized phase II study of sorafenib with or without everolimus in patients with radioactive iodine refractory Hürthle cell thyroid cancer (HCC)(Alliance A091302/ITOG 1706). J. Clin. Oncol. 39, 6076. 10.1200/jco.2021.39.15_suppl.6076

[B32] Siegel R. L. Miller K. D. Jemal A. (2018). Cancer statistics, 2018. Ca. Cancer J. Clin. 68, 7–30. 10.3322/caac.21442 29313949

[B33] Subbiah V. Hu M. I. Wirth L. J. Schuler M. Mansfield A. S. Curigliano G. (2021). Pralsetinib for patients with advanced or metastatic RET-altered thyroid cancer (ARROW): a multi-cohort, open-label, registrational, phase 1/2 study. Lancet. Diabetes Endocrinol. 9, 491–501. 10.1016/S2213-8587(21)00120-0 34118198 PMC13224057

[B34] Villanueva R. A. M. Chen Z. J. (2019). “ggplot2: elegant graphics for data analysis,” in Measurement interdisciplinary research & perspectives. 2nd ed., 17, 160–167.

[B35] Wagle N. Emery C. Berger M. F. Davis M. J. Sawyer A. Pochanard P. (2011). Dissecting therapeutic resistance to RAF inhibition in melanoma by tumor genomic profiling. J. Clin. Oncol. 29, 3085–3096. 10.1200/JCO.2010.33.2312 21383288 PMC3157968

[B36] Wei Y. Royston P. (2017). Reconstructing time-to-event data from published Kaplan-Meier curves. Stata J. 17, 786–802. 10.1177/1536867x1801700402 29398980 PMC5796634

[B37] Wirth L. J. Sherman E. Robinson B. Solomon B. Kang H. Lorch J. (2020). Efficacy of selpercatinib in RET-altered thyroid cancers. N. Engl. J. Med. 383, 825–835. 10.1056/NEJMoa2005651 32846061 PMC10777663

[B38] Zhao Y. Liu J. Cai X. Pan Z. Liu J. Yin W. (2019). Efficacy and safety of first line treatments for patients with advanced epidermal growth factor receptor mutated, non-small cell lung cancer: systematic review and network meta-analysis. BMJ 367, l5460. 10.1136/bmj.l5460 31591158 PMC6778694

[B39] Zheng X. Xu Z. Ji Q. Ge M. Shi F. Qin J. (2021). A randomized, phase III study of lenvatinib in Chinese patients with radioiodine-refractory differentiated thyroid cancer. Clin. Cancer Res. 27, 5502–5509. 10.1158/1078-0432.CCR-21-0761 34326132 PMC9401493

