# Supplementary material for: Efficacy and safety of targeted therapeutics for patients with radioiodine-refractory differentiated thyroid cancer: Systematic review and network meta-analysis
Source: Front Pharmacol. 2022 Aug 26;13:933648. doi: 10.3389/fphar.2022.933648 (PMC9461142; doi:10.3389/fphar.2022.933648)
Supplement: Supplementary file 1 [file DataSheet1.PDF]

Supplementary Material

Ji XY, Liang WL, Lv GX, et al. Efficacy and safety of targeted therapeutics for patients with radioiodine-refractory differentiated thyroid cancer: systematic review and network meta-analysis

Supplementary Table S1. Checklist of the PRISMA extension for network meta-analysis

Supplementary Table S2. Consistency between reported median PFS and derived median PFS from Kaplan-Meier survival curve

Supplementary Table S3. Profile of grade 3 or higher adverse events in each targeted therapeutic option

Supplementary Text. Literature search strategy

Supplementary Figure S1. Convergence of the four chains established by inspection of the Brooks-Gelman-Rubin diagnostic and the trace plot

Supplementary Figure S3. Summary of bias risk assessment of included studies using the Cochrane risk of bias tool

Supplementary Figure S4. Pairwise meta-analysis of PFS and OS in comparison of targeted therapeutics versus placebo in patients with radioiodine-refractory differentiated thyroid cancer using fixed model.

Supplementary Figure S4. Bayesian ranking profiles of comparable treatments on PFS, OS, DCR, and ⩾3 AEs for patients with radioiodine-refractory differentiated thyroid cancer

Supplementary Figure S5. Sensitive analysis of network meta-analysis on PFS, OS, DCR, and ⩾3 AEs by excluding studies with 100% of previously treated with targeted therapy.

Supplementary Table S1. Checklist of the PRISMA extension for network meta-analysis.

Section/topic	Item#	Checklist item*	Reported on page #
**TITLE**			
Title	1	Identify the report as a systematic review *incorporating a network meta- analysis (or related form of meta-analysis).*	1
**ABSTRACT**			
Structured summary	2	Provide a structured summary including, as applicable:<br>• Background: main objectives;<br>• Methods: data sources; study eligibility criteria, participants, and interventions; study appraisal and *synthesis methods, such as network meta- analysis.*<br>• Results: number of studies and participants identified; summary estimates with corresponding confidence/credible intervals; *treatment rankings may also be discussed. Authors may choose to summarize pairwise comparisons against a chosen treatment included in their analyses for brevity.*<br>• Discussion/Conclusions: limitations; conclusions and implications of findings.<br>• Other: primary source of funding; systematic review registration number with registry name.	2
**INTRODUCTION**			
Rationale	3	Describe the rationale for the review in the context of what is already known, *including mention of why a network meta-analysis has been conducted.*	3
Objectives	4	Provide an explicit statement of questions being addressed with reference to participants, interventions, comparisons, outcomes, and study design (PICOS).	3
**METHODS**			
Protocol and registration	5	Indicate if a review protocol exists, if and where it can be accessed (e.g., Web address), and, if available, provide registration information including registration number.	3
Eligibility criteria	6	Specify study characteristics (e.g., PICOS, length of follow-up) and report characteristics (e.g., years considered, language, publication status) used as criteria for eligibility, giving rationale. *Clearly describe eligible treatments included in the treatment network, and note whether any have been clustered or merged into the same node (with justification).*	3-4
Information sources	7	Describe all information sources (e.g., databases with dates of coverage, contact with study authors to identify additional studies) in the search and date last searched.	3
Search	8	Present full electronic search strategy for at least one database, including any limits used, such that it could be repeated.	eText
Study selection	9	State the process for selecting studies (i.e., screening, eligibility, included in systematic review, and, if applicable, included in the meta-analysis).	3-4 (figure 1)
Data collection process	10	Describe method of data extraction from reports (e.g., piloted forms, independently, in duplicate) and any processes for obtaining and confirming data from investigators.	4
Data items	11	List and define all variables for which data were sought (e.g., PICOS, funding sources) and any assumptions and simplifications made.	4
Geometry of the network	S1	Describe methods used to explore the geometry of the treatment network under study and potential biases related to it. This should include how the evidence base has been graphically summarized for presentation, and what characteristics were compiled and used to describe the evidence base to readers.	4
Risk of bias in individual studies	12	Describe methods used for assessing risk of bias of individual studies (including specification of whether this was done at the study or outcome level), and how this information is to be used in any data synthesis.	4
Summary measures	13	State the principal summary measures (e.g., risk ratio, difference in means). *Also describe the use of additional summary measures assessed, such as treatment rankings and surface under the cumulative ranking curve (SUCRA) values, as well as modified approaches used to present summary findings from meta-analyses.*	4-5

Synthesis of results	14	Describe the methods of handling data and combining results of studies for each network meta-analysis. This should include, but not be limited to:<br>• *Handling of multi-arm trials;*<br>• *Selection of variance structure;*<br>• *Selection of prior distributions in Bayesian analyses; and*<br>• *Assessment of model fit.*	4-5
Assessment of Inconsistency	S2	Describe the statistical methods used to evaluate the agreement of direct and indirect evidence in the treatment network(s) studied. Describe efforts taken to address its presence when found.	4-5
Risk of bias across studies	15	Specify any assessment of risk of bias that may affect the cumulative evidence (e.g., publication bias, selective reporting within studies).	4
Additional analyses	16	Describe methods of additional analyses, if done, indicating which were pre- specified. This may include, but not be limited to the following:<br>• Sensitivity or subgroup analyses;<br>• Meta-regression analyses;<br>• *Alternative formulations of the treatment network; and*<br>• *Use of alternative prior distributions for Bayesian analyses (if applicable).*	5
**RESULTS**			
Study selection	17	Give numbers of studies screened, assessed for eligibility, and included in the review, with reasons for exclusions at each stage, ideally with a flow diagram.	5 (figure 1)
Presentation of network structure	S3	Provide a network graph of the included studies to enable visualization of the geometry of the treatment network	5 (figure 2)
Summary of network geometry	S4	Provide a brief overview of characteristics of the treatment network. This may include commentary on the abundance of trials and randomized patients for the different interventions and pairwise comparisons in the network, gaps of evidence in the treatment network, and potential biases reflected by the network structure.	5
Study characteristics	18	For each study, present characteristics for which data were extracted (e.g., study size, PICOS, follow-up period) and provide the citations.	5 (table 1)
Risk of bias within studies	19	Present data on risk of bias of each study and, if available, any outcome level assessment.	eFigure 2
Results of individual studies	20	For all outcomes considered (benefits or harms), present, for each study:1) simple summary data for each intervention group, and 2) effect estimates and confidence/credible intervals. *Modified approaches may be needed to deal with information from larger networks.*	5-6
Synthesis of results	21	Present results of each meta-analysis done, including confidence/credible intervals. *In larger networks, authors may focus on comparisons versus a particular comparator (e.g. placebo or standard care), with full findings presented in an appendix. League tables and forest plots may be considered to summarize pairwise comparisons.* If additional summary measures were explored (such as treatment rankings), these should also be presented.	5-6, Figure 2 and 3
Exploration for inconsistency	S5	Describe results from investigations of inconsistency. This may include such information as measures of model fit to compare consistency and inconsistency models, *P* values from statistical tests, or summary of inconsistency estimates from different parts of the treatment network.	4
Risk of bias across studies	22	Present results of any assessment of risk of bias across studies.	5
Additional analysis	23	Give results of additional analyses, if done (e.g., sensitivity or subgroup analyses, meta-regression, *alternative network geometries studied, alternative choice of prior distributions for Bayesian analyses,* and so forth.	6

**DISCUSSION**			
Summary of evidence	24	Summarize the main findings including the strength of evidence for each main outcome; consider their relevance to key groups (e.g., healthcare providers, users, and policy makers).	6-8
Limitations	25	Discuss limitations at study and outcome level (e.g., risk of bias), and at review-level (e.g., incomplete retrieval of identified research, reporting bias). *Comment on the validity of the assumptions, such as transitivity and consistency. Comment on any concerns regarding network geometry (e.g., avoidance of certain comparisons).*	8
Conclusions	26	Provide a general interpretation of the results in the context of other evidence, and implications for future research.	8
FUNDING			
Funding	27	Describe sources of funding for the systematic review and other support (e.g., supply of data); role of funders for the systematic review.	9

PICOS = population, intervention, comparators, outcomes, study design.
*Text in italics indicates wording specific to reporting of network meta-analyses that has been added to guidance from the PRISMA statement.
† Authors may wish to plan for use of appendices to present all relevant information in full detail for items in this section.

Supplementary Table S2. Consistency between reported median PFS and derived median PFS from Kaplan-Meier survival curve

Study	Treatment	Control	Reported median PFS		Derived median PFS from Kaplan-Meier survival curve	
			Treatment	Control	Treatment	Control
Schlumberger 2015	Lenvatinib	Placebo	18.3 (15.1-NE)	3.6 (2.2-3.7)	18.3 (15.0-NE)	3.6 (2.4-3.8)
Zheng 2021	Lenvatinib	Placebo	23.9 (12.9-NE)	3.7 (1.9-5.6)	23.9 (12.9-NE)	3.8 (1.9-6.2)
Lin 2021	Apatinib	Placebo	22.2 (10.91-NE)	4.5 (1.94-9.17)	22.2 (11.1-NE)	4.5 (2.0-9.2)
Brose 2021	Cabozantinib	Placebo	11	1.9	11.1 (7.4-NE)	2.0 (1.9-3.7)
Brose 2014	Sorafenib	Placebo	10.8	5.8	11.0 (9.3-13.1)	5.9 (5.4-7.9)
Leboulleux 2012	Vandetanib	Placebo	11.1 (7.7-14.0)	5.9 (4.0-8.9)	11.0 (7.9-13.9)	5.8 (3.9-11.0)
Brose 2020	Lenvatinib (LD)	Lenvatinib	24.4 (14.7-NE)	NR	24.4 (16.9-NE)	NR
Sherman 2021	Sorafenib plus everolimus	Sorafenib	24.7 (6.1-33.8)	10.9 (5.5-29.7)	24.8 (6.2-33.8)	10.9 (5.6-36.4)

PFS, progression-free survival; NE, not estimable; NR, not reached

Supplementary Table S3. Profile of grade 3 or higher adverse events in each targeted therapeutic option

	Lenvatinib (n=364)	Apatinib (n=46)	Cabozantinib (n=125)	Sorafenib (n=207)	Vandetanib (n=73)	Nintedanib (n=45)	Donafenib (200mg) (n=17)	Donafenib (300mg) (n=18)	Pazopanib (continuous) (n=50)	Pazopanib (intermittent) (n=50)	Placebo (n=593)
Any grade ≥ 3 AEs	288 (79.1)	36 (78.2)	80 (64.0)	133 (64.3)	39 (53.4)	23 (46.0)	-	-	29 (58.0)	32 (64.0)	104 (17.5)
Grade ≥ 3 treatment-related AEs	286 (78.6)	34 (73.9)	-	-	-	-	9 (52.9)	9 (50.0)	17 (34.0)	19 (38.0)	21 (3.5)
Serious AEs	40 (11.0)	-	-	77 (37.2)	-	-	-	-	-	-	71 (12.0)
Serious treatment-related AEs	-	-	20 (16.0)	-	-	-	1 (5.9)	1 (5.6)	11 (22.0)	10 (20.0)	1 (0.2)
Fatal related to AEs	16 (4.4)	0	0	1 (0.5)	2 (2.7)	-	-	-	0	3 (6.0)	5 (0.8)
Hand-foot syndrome	19 (5.2)	8 (17.4)	13 (10.4)	42 (20.3)	-	-	2 (11.8)	2 (11.1)	0	0	0
Hypertension	173 (47.5)	16 (34.8)	11 (8.8)	20 (9.7)	-	4 (8.9)	4 (23.5)	4 (22.2)	2 (4.0)	7 (14.0)	16 (2.7)
Proteinuria	50 (13.7)	7 (15.2)	1 (0.8)	-	-	-	0	0	-	-	0
Diarrhoea	28 (7.7)	7 (15.2)	9 (7.2)	12 (5.8)	7 (9.6)	4 (8.9)	0	0	1 (2.0)	5 (10.0)	3 (0.5)
Asthenia	-	0	3 (2.4)	-	5 (6.8)	-	0	0	3 (6.0)	1 (2.0)	3 (0.5)
Hypocalcaemia	11 (3.0)	3 (6.5)	9 (7.2)	19 (9.2)	3 (4.1)	-	-	-	0	0	5 (0.8)
Platelet count decreased	7 (1.9)	0	-	-	-	-	-	-	0	0	0
Blood lactate dehydrogenase increased	0	0	-	-	-	-	-	-	-	-	0
Headache	7 (1.9)	1 (2.2)	2 (1.6)	0	-	-	-	-	0	0	0
Blood bilirubin increased	-	1 (2.2)	-	-	-	-	-	-	0	0	0
Neutrophil count decreased	-	0	2 (1.6)	-	-	-	-	-	1 (2.0)	0	0
White blood cell count decreased	-	0	1 (0.8)	-	-	-	-	-	-	-	0
Hypokalaemia	-	1 (2.2)	1 (0.8)	-	3 (4.1)	-	-	-	-	-	1 (0.2)
Cough	0	1 (2.2)	0	0	-	-	-	-	-	-	0
Aspartate aminotransferase increased	-	0	0	2 (1.0)	-	-	-	-	1 (2.0)	0	0
Gamma-glutamyltransferase increased	-	1 (2.2)	2 (1.6)	-	-	-	-	-	0	2 (4.0)	0
Decreased appetite	16 (4.4)	1 (2.2)	4 (3.2)	-	1 (1.4)	-	-	-	1 (2.0)	2 (4.0)	2 (0.3)
Blood urine present	-	1 (2.2)	-	-	-	-	-	-	-	-	0
Hematuria	-	0	-	-	-	-	-	-	-	-	0
Alanine aminotransferase increased	-	1 (2.2)	1 (0.8)	6 (2.9)	-	-	-	-	1 (2.0)	0	0
Hyperglycaemia	-	0	-	-	-	-	-	-	-	-	0
Weight decreased	28 (7.7)	1 (2.2)	1 (0.8)	12 (5.8)	-	1 (2.2)	-	-	0	0	2 (0.3)
Electrocardiogram QT prolonged	-	1 (2.2)	-	-	10 (13.7)	-	-	-	-	-	0
Blood alkaline phosphatase increased	-	0	1 (0.8)	-	-	-	-	-	0	0	0
Haematochezia	-	0	-	-	-	-	-	-	-	-	0
Anemia	-	0	-	-	-	1 (2.2)	-	-	-	-	1 (0.2)
Dysphonia	3 (0.8)	-	0	-	-	-	-	-	-	-	0
Bilirubin conjugated increased	-	0	-	-	-	-	-	-	-	-	0
Stomatitis	11 (3.0)	1 (2.2)	3 (2.4)	-	-	-	0	0	0	0	0
Dyspnoea	-	-	4 (3.2)	10 (4.8)	2 (2.7)	-	-	-	-	-	11 (1.9)
Nausea	6 (1.6)	-	4 (3.2)	0	-	2 (4.4)	-	-	0	1 (2.0)	1 (0.2)
Fatigue	24 (6.6)	-	10 (8.0)	12 (5.8)	4 (5.5)	3 (6.7)	-	-	0	2 (4.0)	7 (1.2)
Vomiting	5 (1.4)	-	1 (0.8)	1 (0.5)	-	-	-	-	0	0	0
Mucosal inflammation	-	-	3 (2.4)	-	-	-	-	-	-	-	0
Hypomagnesaemia	-	-	1 (0.8)	-	-	-	-	-	0	0	0
Constipation	1 (0.3)	-	0	0	-	-	-	-	-	-	1 (0.2)
Dry mouth	1 (0.3)	-	1 (0.8)	-	-	-	-	-	-	-	0
Arthralgia	0	-	2 (1.6)	-	-	-	0	0	-	-	0
Abdominal pain	1 (0.3)	-	2 (1.6)	3 (1.4)	-	1 (2.2)	-	-	0	0	2 (0.3)
Anaemia	-	-	2 (1.6)	-	-	-	-	-	-	-	0
Pain in extremity	-	-	1 (0.8)	-	-	-	-	-	-	-	0
Back pain	-	-	1 (0.8)	2 (1.0)	-	-	-	-	-	-	3 (0.5)
Leukopenia	-	-	1 (0.8)	-	-	-	-	-	-	-	0
Pain	-	-	1 (0.8)	-	-	-	-	-	-	-	1 (0.2)
Pulmonary embolism	7 (1.9)	-	4 (3.2)	-	-	-	-	-	-	-	2 (0.3)
Amylase increased	-	-	1 (0.8)	-	-	-	-	-	-	-	1 (0.2)
Chest pain	-	-	2 (1.6)	-	-	-	-	-	-	-	0
Dysphagia	-	-	1 (0.8)	-	-	-	-	-	-	-	0
Hair colour changes	-	-	1 (0.8)	-	-	-	-	-	0	0	0
Neutropenia	-	-	1 (0.8)	-	-	-	-	-	-	-	0
Pleural effusion	-	-	4 (3.2)	-	-	-	-	-	-	-	2 (0.3)
Pneumonia	-	-	2 (1.6)	-	-	-	-	-	-	-	0
Hyponatraemia	-	-	1 (0.8)	-	-	-	-	-	-	-	1 (0.2)
Insomnia	-	-	0	-	-	-	-	-	-	-	1 (0.2)
Musculoskeletal pain	-	-	1 (0.8)	-	-	-	-	-	-	-	1 (0.2)
Neck pain	-	-	1 (0.8)	-	-	-	-	-	-	-	1 (0.2)
Pruritus	-	-	0	2 (1.0)	-	-	-	-	-	-	1 (0.2)
Skin ulcer	-	-	2 (1.6)	-	-	-	-	-	-	-	0
Confusional state	-	-	1 (0.8)	-	-	-	-	-	-	-	0
Deep vein thrombosis	-	-	1 (0.8)	-	-	-	-	-	-	-	0
Dehydration	-	-	2 (1.6)	-	-	-	-	-	-	-	0
Disease progression	-	-	3 (2.4)	-	-	-	-	-	-	-	2 (0.3)
Fall	-	-	0	-	-	-	-	-	-	-	1 (0.2)
Electrolyte imbalance	-	-	1 (0.8)	-	-	-	-	-	-	-	0
Gastro-oesophageal reflux disease	-	-	1 (0.8)	-	-	-	-	-	-	-	0
General physical health deterioration	-	-	2 (2%)	-	-	-	-	-	-	-	1 (0.2)
Haemoptysis	-	-	1 (0.8)	-	-	-	-	-	-	-	0
Hypoalbuminaemia	-	-	1 (0.8)	-	-	-	-	-	-	-	0
Painful respiration	-	-	1 (0.8)	-	-	-	-	-	-	-	0
Blood magnesium decreased	-	-	1 (0.8)	-	-	-	-	-	-	-	0
Haematoma	-	-	1 (0.8)	-	-	-	-	-	-	-	0
Hypercalcaemia	-	-	1 (0.8)	-	-	-	-	-	-	-	2 (0.3)
Hypertensive crisis	-	-	1 (0.8)	-	-	-	-	-	-	-	0
Intestinal obstruction	-	-	1 (0.8)	-	-	-	-	-	-	-	0

Jaundice cholestatic	-	-	1 (0.8)	-	-	-	-	-	-	-	0
Large-intestine perforation	-	-	1 (0.8)	-	-	-	-	-	-	-	0
Laryngeal necrosis	-	-	1 (0.8)	-	-	-	-	-	-	-	0
Lung disorder	-	-	1 (0.8)	-	-	-	-	-	-	-	0
Oesophageal stenosis	-	-	1 (0.8)	-	-	-	-	-	-	-	0
Osteonecrosis of jaw	-	-	1 (0.8)	-	-	-	-	-	-	-	0
Pathological fracture	-	-	1 (0.8)	-	-	-	-	-	-	-	0
Spinal cord compression	-	-	1 (0.8)	-	-	-	-	-	-	-	1 (0.2)
Acute kidney injury	-	-	1 (0.8)	-	-	-	-	-	-	-	0
Anal abscess	-	-	1 (0.8)	-	-	-	-	-	-	-	0
Aptyalism	-	-	1 (0.8)	-	-	-	-	-	-	-	0
Arterial haemorrhage	-	-	1 (0.8)	-	-	-	-	-	-	-	0
Arthropod bite	-	-	1 (0.8)	-	-	-	-	-	-	-	0
Atrial fibrillation	-	-	1 (0.8)	-	-	-	-	-	-	-	0
Bone lesion	-	-	1 (0.8)	-	-	-	-	-	-	-	0
Cancer pain	-	-	1 (0.8)	-	-	-	-	-	-	-	0
Cardiac arrest	-	-	1 (0.8)	-	-	-	-	-	-	-	1 (0.2)
Cardio-respiratory arrest	-	-	1 (0.8)	-	-	-	-	-	-	-	0
Cholangitis	-	-	1 (0.8)	-	-	-	-	-	-	-	0
Cholangitis acute	-	-	1 (0.8)	-	-	-	-	-	-	-	0
Cholelithiasis	-	-	1 (0.8)	-	-	-	-	-	-	-	0
COVID-19	-	-	1 (0.8)	-	-	-	-	-	-	-	0
Ejection fraction decreased	-	-	1 (0.8)	-	-	-	-	-	-	-	0
Radicular pain	-	-	1 (0.8)	-	-	-	-	-	-	-	0
Rectal abscess	-	-	1 (0.8)	-	-	-	-	-	-	-	0
Renal impairment	-	-	1 (0.8)	-	-	-	-	-	-	-	0
Spinal fracture	-	-	0	-	-	-	-	-	-	-	1 (0.2)
Syncope	-	-	1 (0.8)	-	-	-	-	-	-	-	0
Thyroid cancer	-	-	1 (0.8)	-	-	-	-	-	-	-	1 (0.2)
Thyroid cancer metastatic	-	-	1 (0.8)	-	-	-	-	-	-	-	0
Tumour pain	-	-	0	-	-	-	-	-	-	-	1 (0.2)
Urine output decreased	-	-	1 (0.8)	-	-	-	-	-	-	-	0
Wound dehiscence	-	-	1 (0.8)	-	-	-	-	-	-	-	0
Wound infection	-	-	1 (0.8)	-	-	-	-	-	-	-	0
Carotid artery stenosis	-	-	0	-	-	-	-	-	-	-	1 (0.2)
Hydrothorax	-	-	0	-	-	-	-	-	-	-	1 (0.2)
Lower respiratory tract infection	-	-	0	-	-	-	-	-	-	-	1 (0.2)
Pain in jaw	-	-	0	-	-	-	-	-	-	-	1 (0.2)
Renal failure	-	-	1 (0.8)	-	-	-	-	-	-	-	0
Cerebrovascular accident	-	-	0	-	-	-	-	-	-	-	1 (0.2)
Poorly differentiated thyroid carcinoma	-	-	0	-	-	-	-	-	-	-	1 (0.2)
Hemoglobin decrease	-	-	-	-	-	-	-	-	1 (2.0)	0	-
Abdominal pain upper	-	-	-	-	-	-	-	-	0	1 (2.0)	-
leukocytes decreased	-	-	-	-	-	-	-	-	1 (2.0)	0	-
Lymphocytes decreased	-	-	-	-	-	-	-	-	3 (6.0)	1 (2.0)	-
Muscle spasms	-	-	-	-	-	-	-	-	0	0	-
Dysgeusia	0	-	-	-	-	-	-	-	0	0	0
Alopecia	0	-	-	-	-	-	0	0	-	-	0
Rash	1 (0.3)	-	-	10 (4.8)	-	-	0	0	-	-	0
Hypophosphatemia	-	-	-	-	-	-	0	0	-	-	-
Oropharyngeal pain	1 (0.3)	-	-	-	-	-	1 (5.9)	0	-	-	0
Anorexia	-	-	-	5 (2.4)	-	5 (11.1)	-	-	-	-	0
Lymphopenia	-	-	-	-	-	3 (6.7)	-	-	-	-	0
Myalgia	4 (1.1)	-	-	-	-	-	-	-	-	-	0
Upper abdominal pain	0	-	-	-	-	-	-	-	-	-	0
Peripheral edema	1 (0.3)	-	-	-	-	-	-	-	-	-	0
Dyspepsia	0	-	-	-	-	-	-	-	-	-	0
Oral mucositis	-	-	-	2 (1.0)	-	-	-	-	-	-	0
Neuropathy: sensory	-	-	-	2 (1.0)	-	-	-	-	-	-	0
Pain, extremity – limb	-	-	-	1 (0.5)	-	-	-	-	-	-	1 (0.2)
Dermatology - Other	-	-	-	2 (1.0)	-	-	-	-	-	-	0
voice change	-	-	-	1 (0.5)	-	-	-	-	-	-	0
Fever	-	-	-	3 (1.5)	-	-	-	-	-	-	0
Pain, other	-	-	-	1 (0.5)	-	-	-	-	-	-	1 (0.2)
Pain, throat/pharynx/larynx	-	-	-	0	-	-	-	-	-	-	0
Metabolic/laboratory – other a	-	-	-	0	-	-	-	-	-	-	0
serum TSH increase	-	-	-	0	-	-	-	-	-	-	0
Photosensitivity reaction	-	-	-	-	3 (4.1)	-	-	-	-	-	0.00

Supplementary Text. Literature search strategy

PubMed

((((((((Radioactive iodine–refractory differentiated thyroid cancer[Title/Abstract]) OR (Radioactive iodine refractory thyroid cancer[Title/Abstract])) OR (radioiodine-refractory differentiated thyroid cancer[Title/Abstract])) OR (Radioactive iodine refractory thyroid cancer[Title/Abstract])) OR (Radioiodine refractory differentiated thyroid carcinoma[Title/Abstract])) OR (Radioiodine refractory advanced DTC[Title/Abstract])) OR (Radioiodine-Refractory Thyroid Cancer[Title/Abstract])) OR (RR-DTC[Title/Abstract])) OR (RAIR-DTC[Title/Abstract]) OR ((((((((((Apatinib[Title/Abstract]) OR (Cabozantinib[Title/Abstract])) OR (Lenvatinib[Title/Abstract])) OR (sorafenib[Title/Abstract])) OR (pazopanib[Title/Abstract])) OR (Donafenib[Title/Abstract])) OR (Anlotinib[Title/Abstract])) OR (Nintedanib[Title/Abstract])) OR (Vandetanib[Title/Abstract]))) AND ((Thyroid cancer[Title/Abstract]) OR Thyroid carcinoma[Title/Abstract]))

Embase

('thyroid cancer'/exp OR 'thyroid cancer') AND ('iodine refractory' OR (('iodine'/exp OR iodine) AND refractory)) AND ([controlled clinical trial]/lim OR [randomized controlled trial]/lim) AND ([adult]/lim OR [aged]/lim OR [very elderly]/lim) AND [humans]/lim AND [english]/lim AND [abstracts]/lim AND [clinical study]/lim

ClinicalTrials.gov

Iodine refractory | Completed, terminated, Unknown status studies| thyroid cancer | Adult, Older Adult
also searched for Thyroid Neoplasm, Neoplasm, and Thyroid carcinoma

Supplementary Figure S1. Convergence of the four chains established by inspection of the Brooks-Gelman-Rubin diagnostic and the trace plot.

Brooks-Gelman-Rubin diagnostic for progression-free survival.

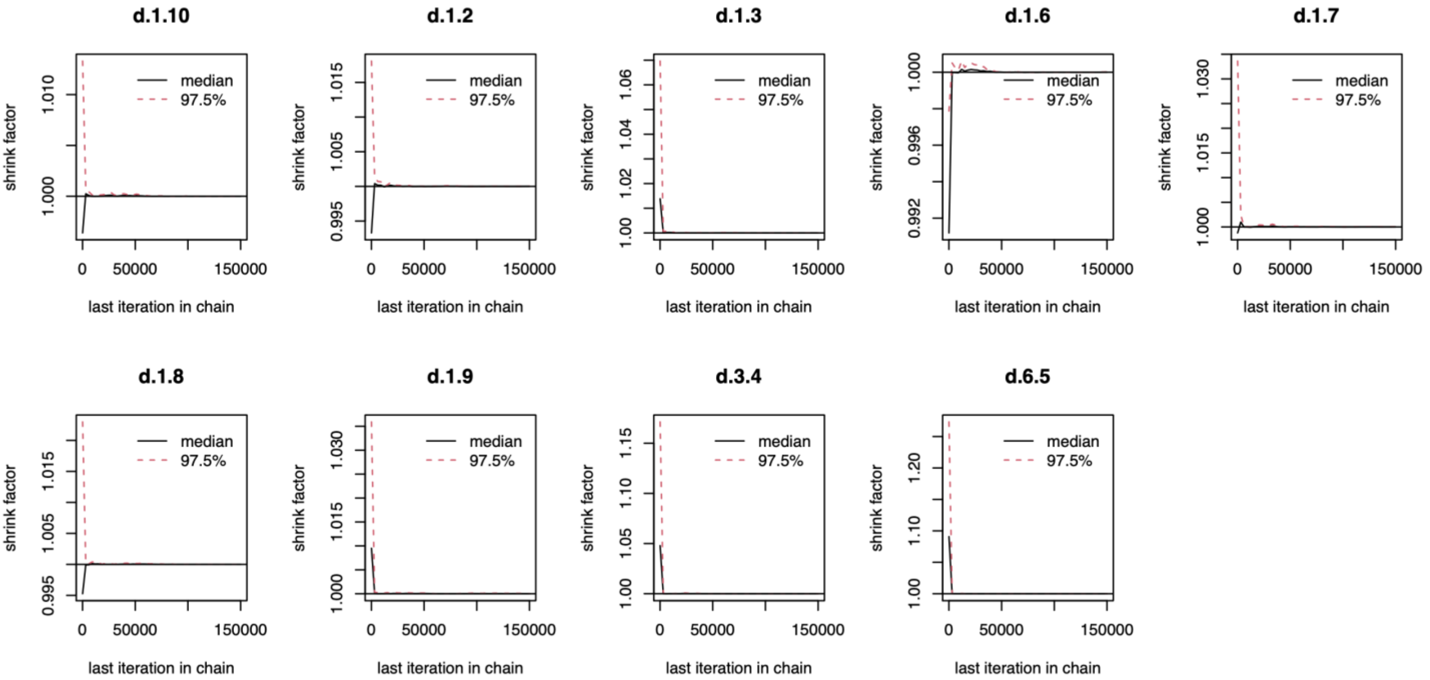

Trace plot for progression-free survival.

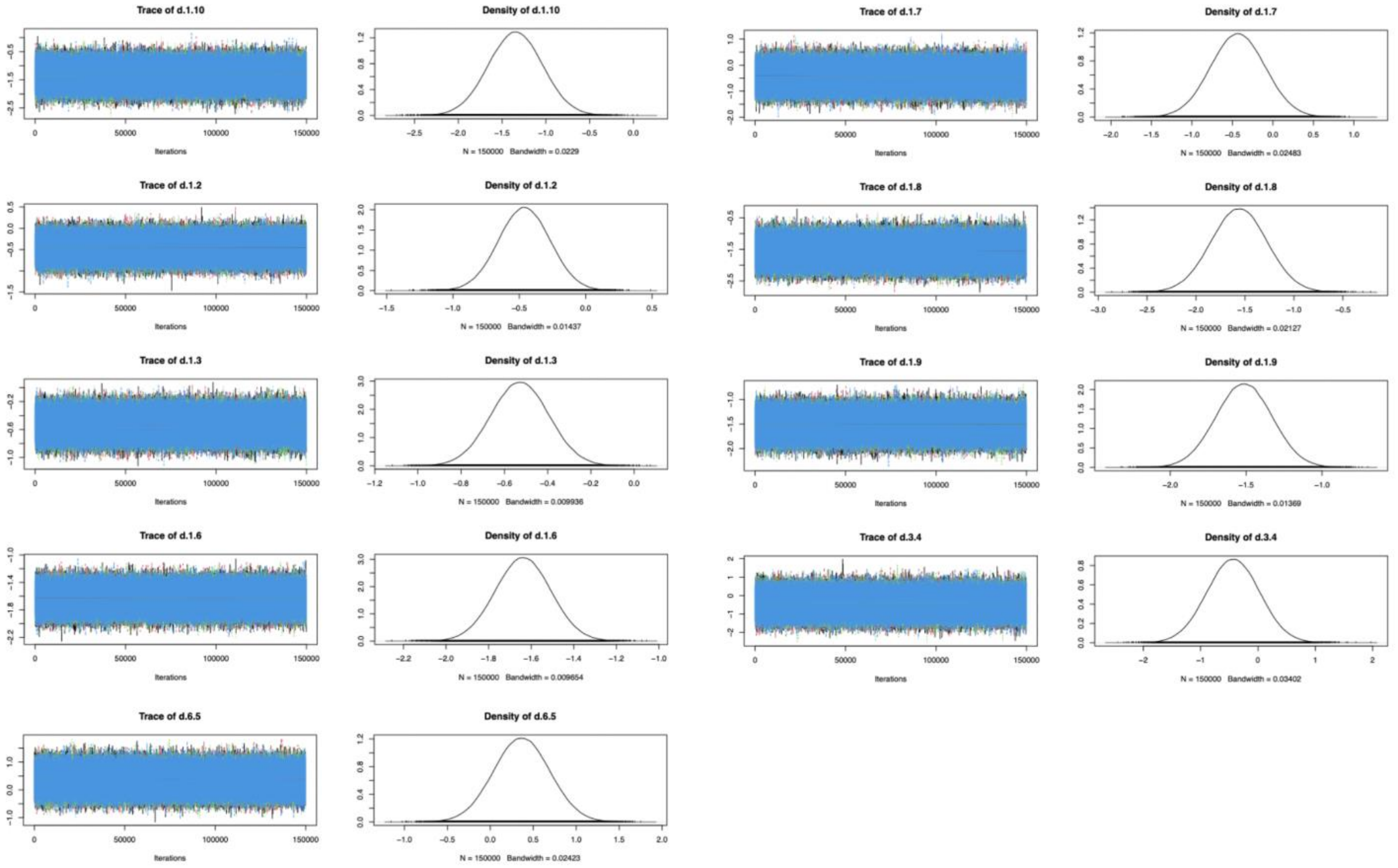

Brooks-Gelman-Rubin diagnostic for overall survival.

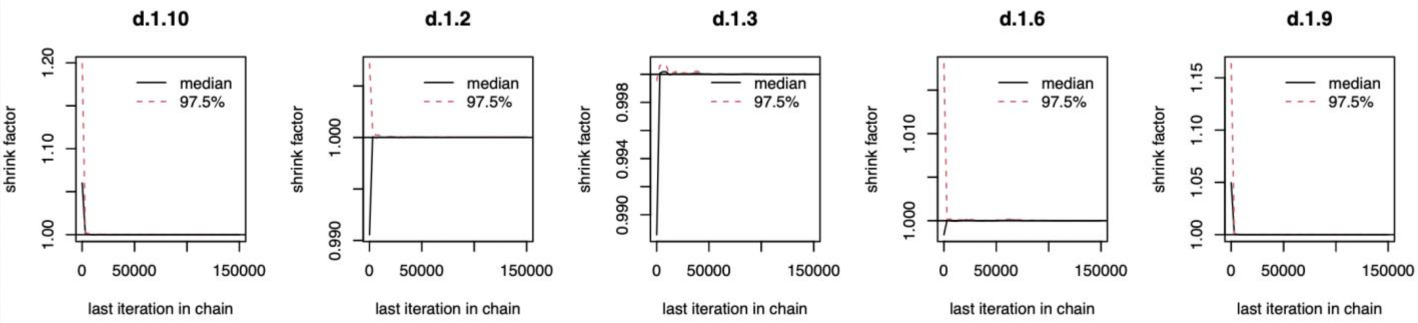

Trace plot for overall survival.

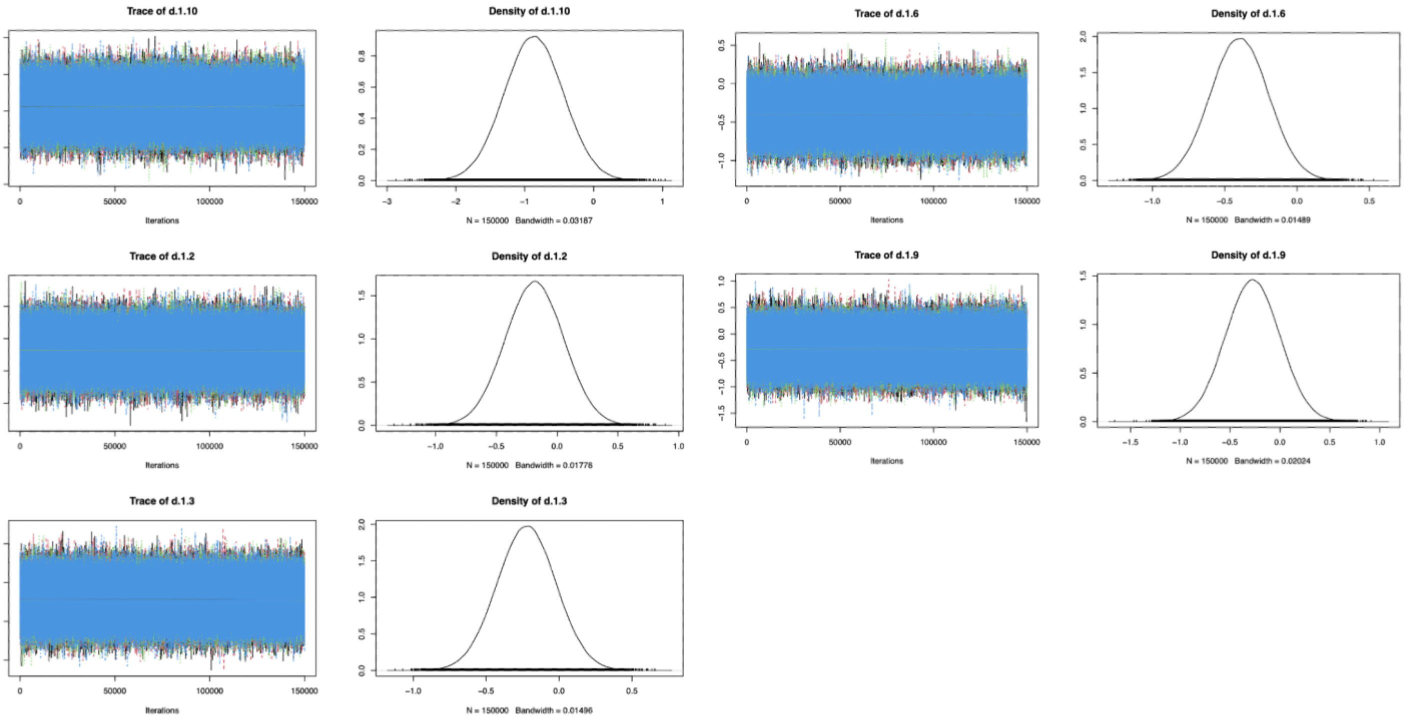

Brooks-Gelman-Rubin diagnostic for disease control rate.

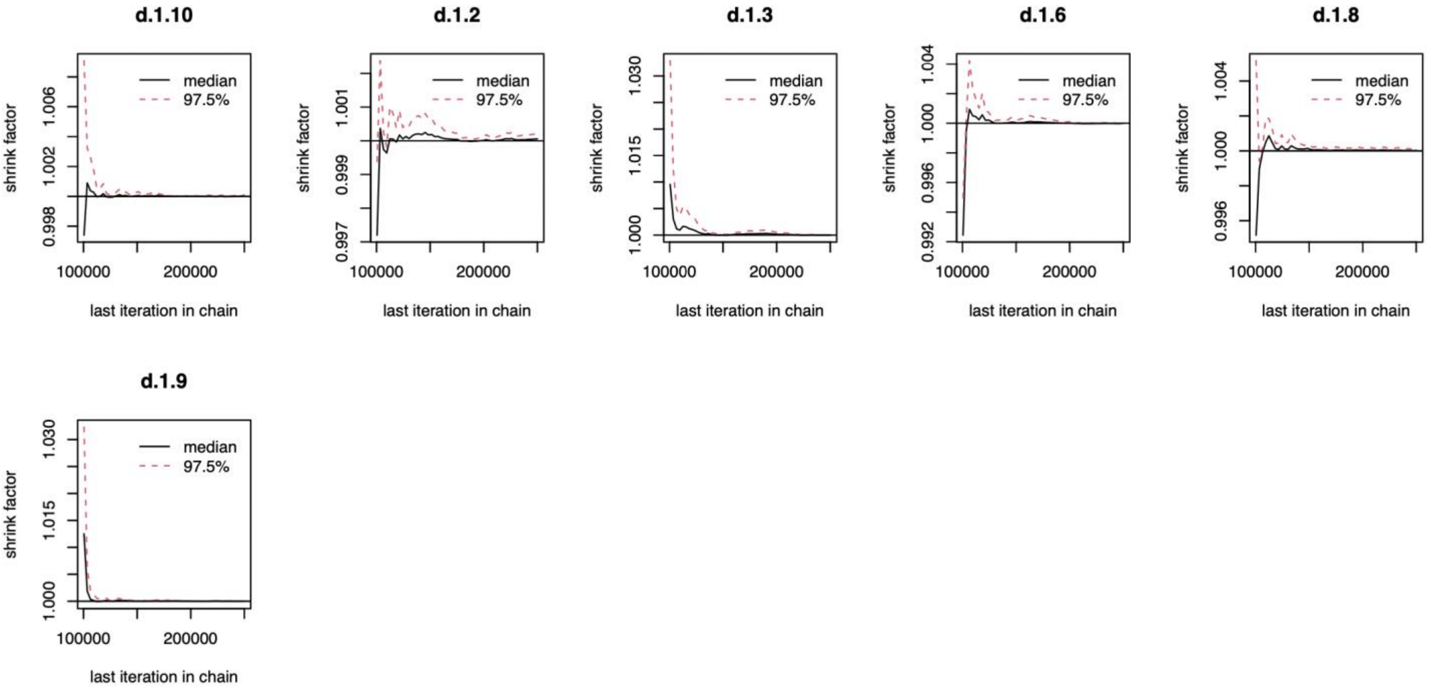

Trace plot for disease control rate.

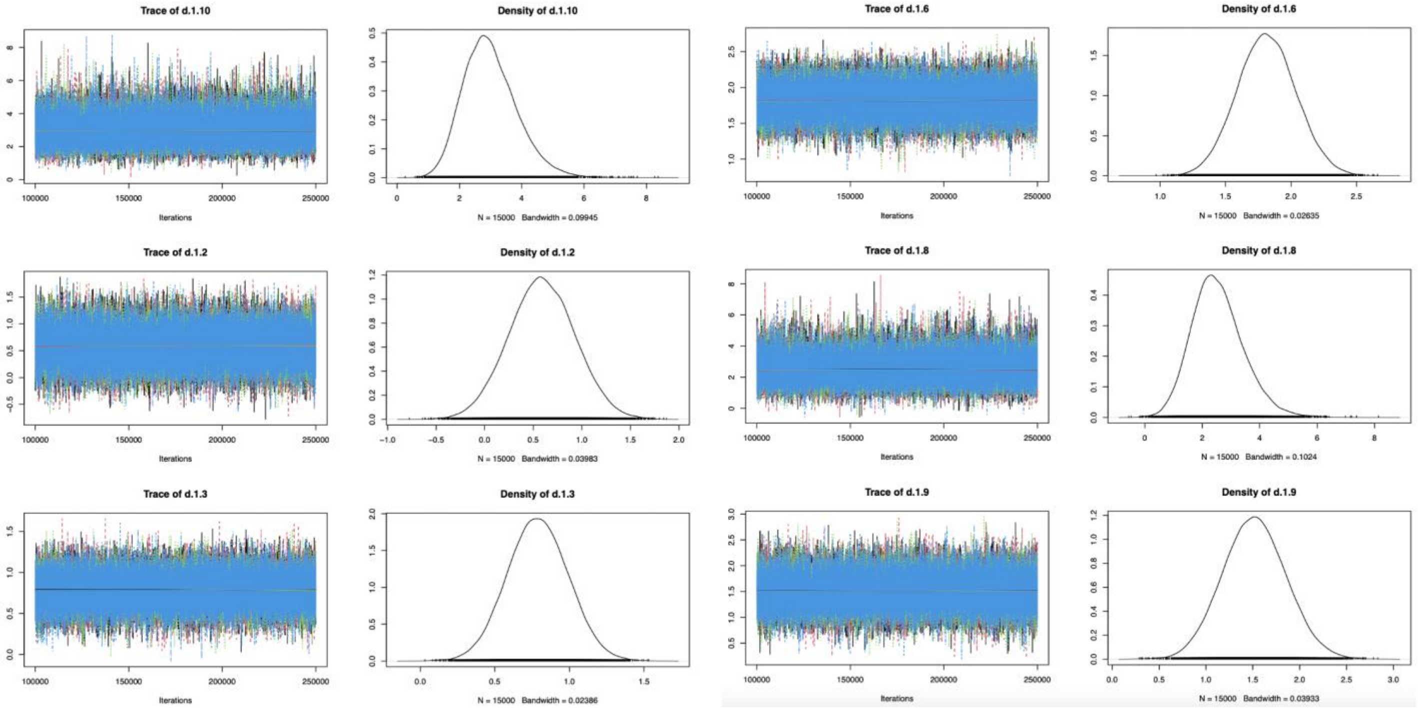

Brooks-Gelman-Rubin diagnostic for grade 3 or higher adverse events.

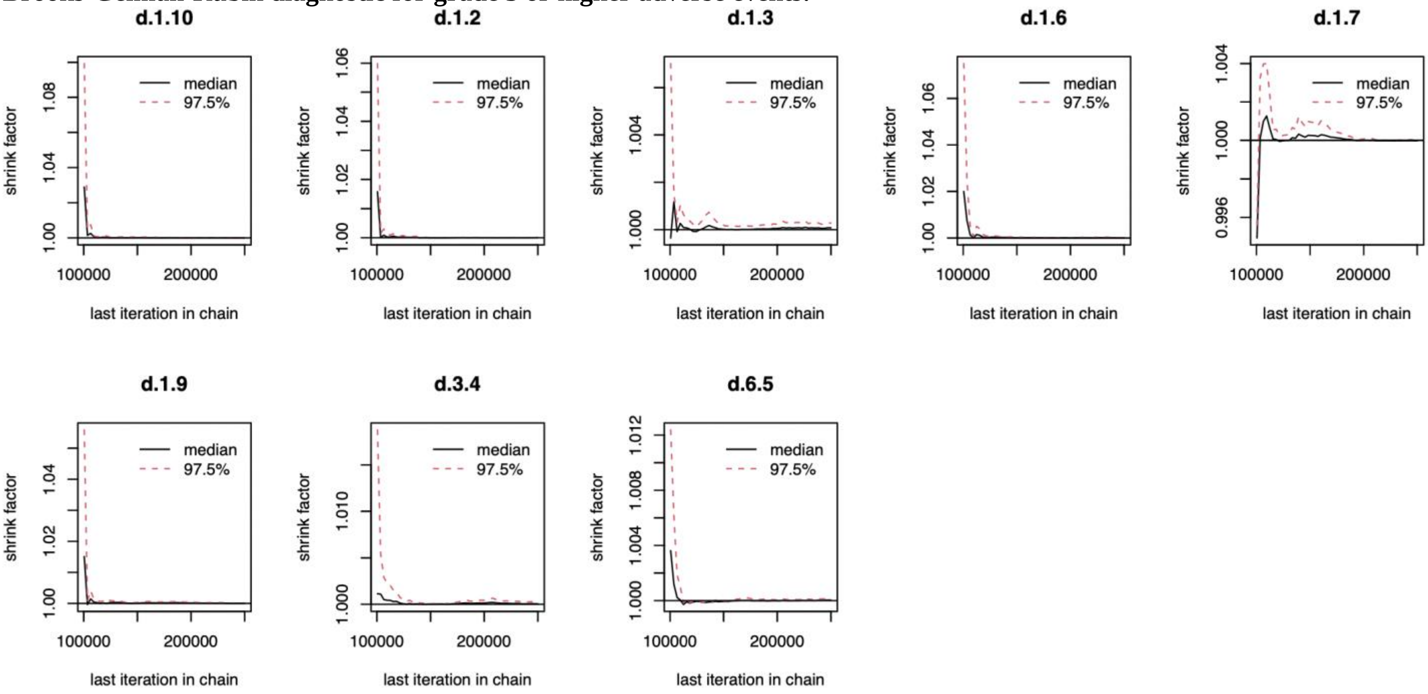

Trace plot for grade 3 or higher adverse events.

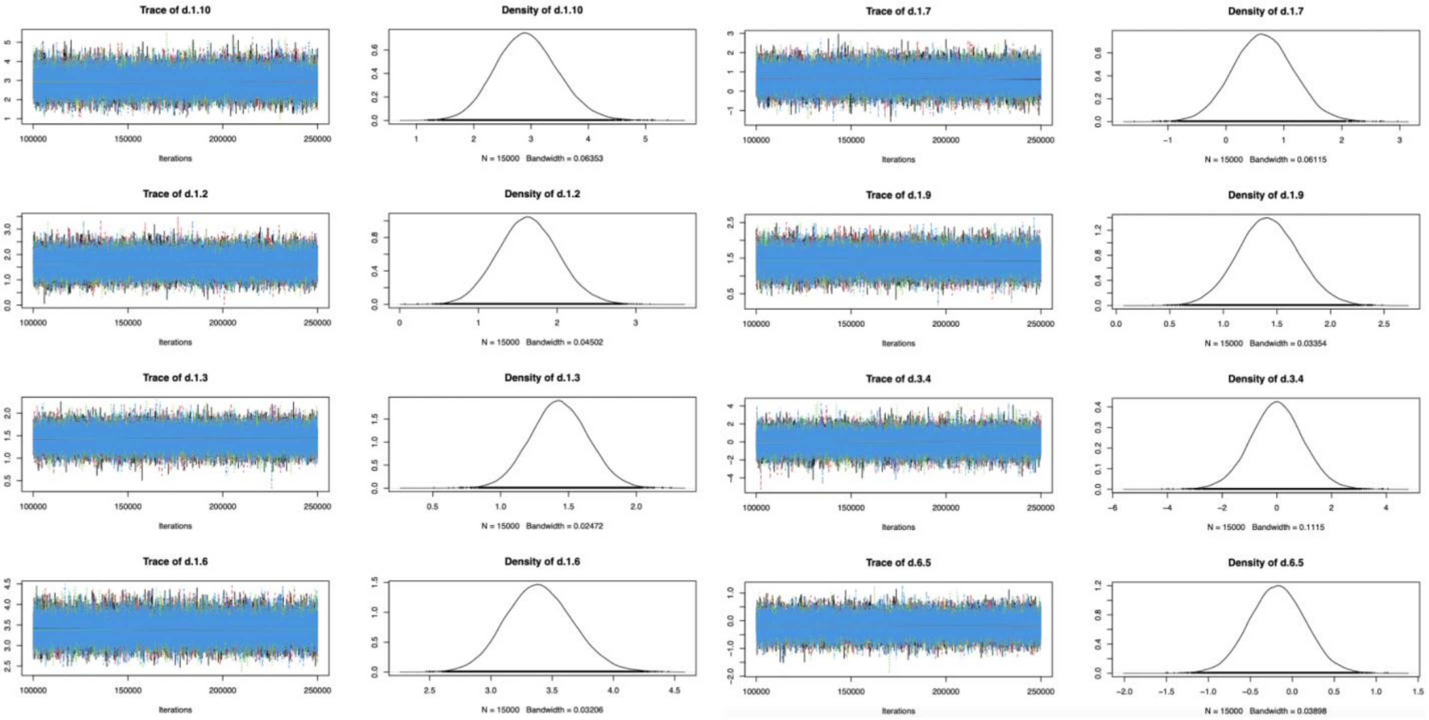

Supplementary Figure S2. Summary of bias risk assessment of included studies using the Cochrane risk of bias tool.

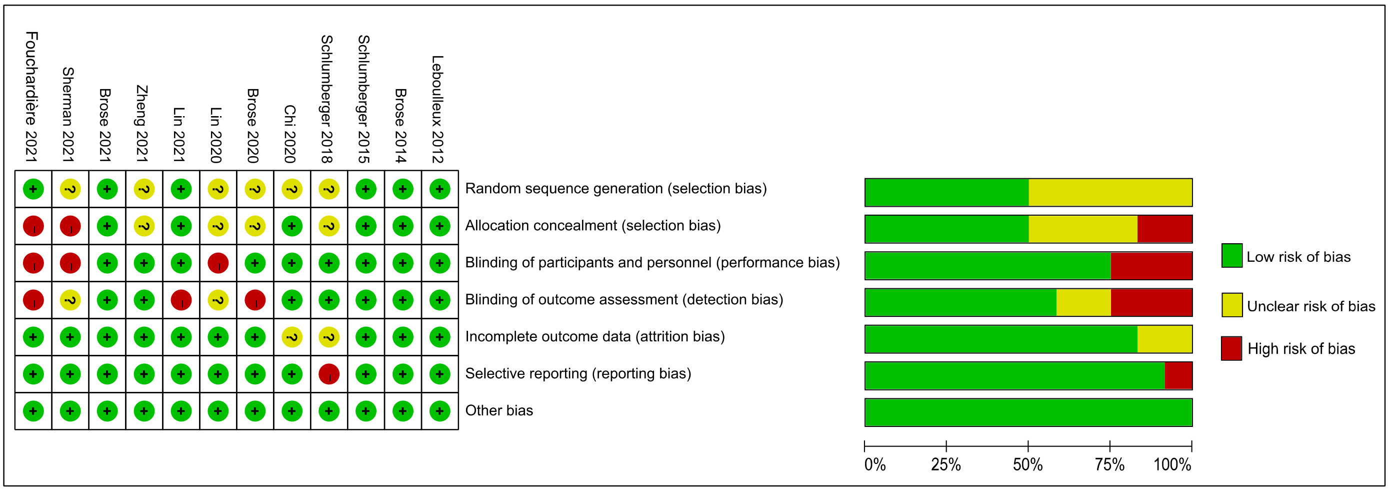

Supplementary Figure S3. Pairwise meta-analysis of PFS and OS in comparison of targeted therapeutics versus placebo in patients with radioiodine-refractory differentiated thyroid cancer using fixed model.

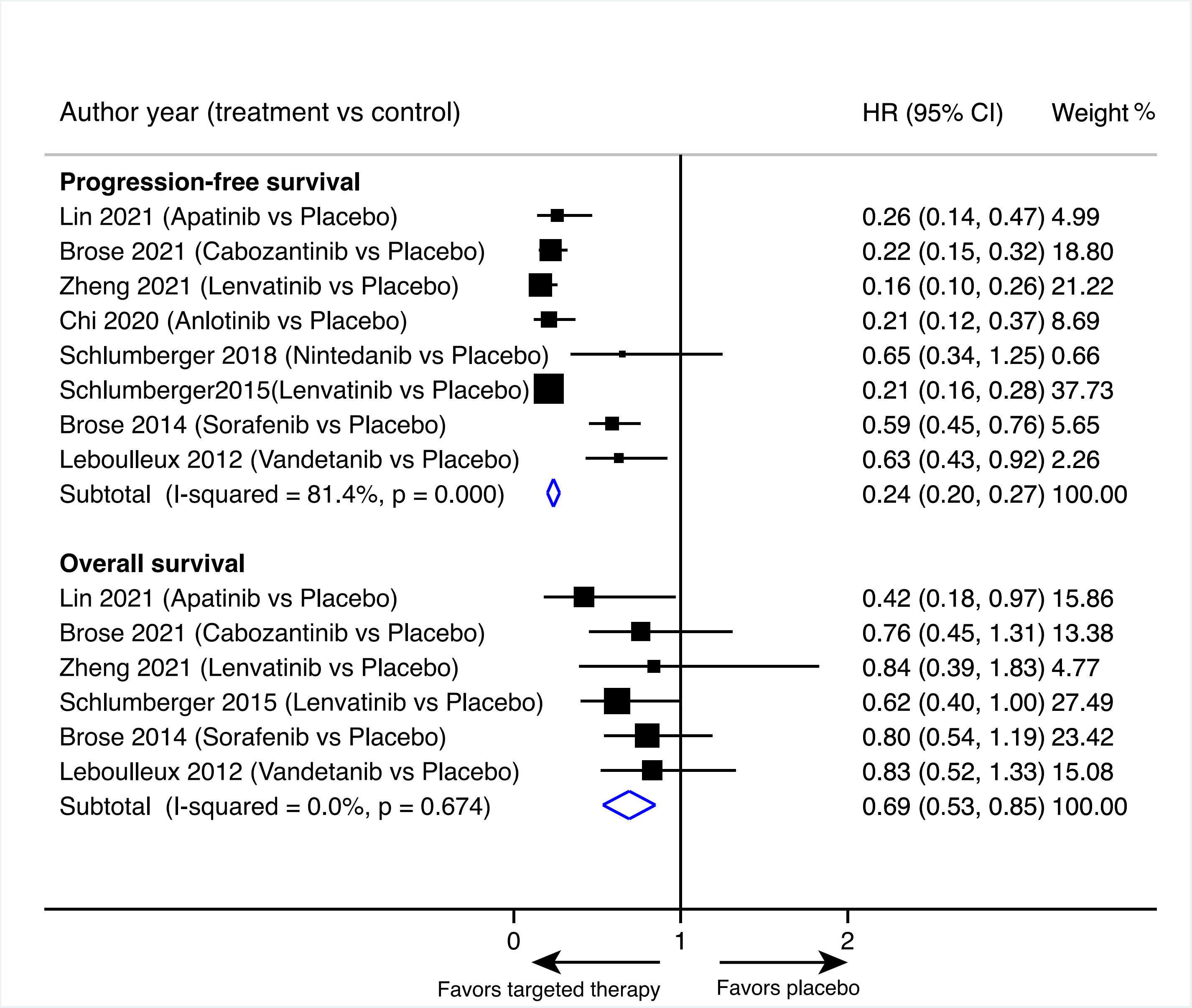

Supplementary Figure S4. Bayesian ranking profiles of comparable treatments on PFS, OS, DCR, and ⩾3 AEs for patients with radioiodine-refractory differentiated thyroid cancer.

	Primary analysis				Sensitive analysis			
	PFS	OS	DCR	⩾3 AEs	PFS	OS	DCR	⩾3 AEs
Lenvatinib	0.88	0.65	0.68	0.93	0.88	0.65	0.66	0.91
Anlotinib	0.80		0.83		0.81		0.82	
Cabozantinib	0.78	0.49	0.56	0.40				
Apatinib	0.68	0.90	0.92	0.80	0.69	0.91	0.91	0.74
Lenvatinib(LD)	0.64			0.86	0.64			0.82
Sorafenib plus Everolimus	0.49			0.41	0.49			0.34
Sorafenib	0.27	0.44	0.29	0.41	0.26	0.44	0.34	0.30
Vandetanib	0.23	0.39	0.22	0.49	0.23	0.39	0.26	0.38
Nintedanib	0.22			0.17				
Placebo	0.01	0.11	0.01	0.02	0.00	0.10	0.01	0.01

Number (percentage of surface under the cumulative ranking curve (SUCRA)) in each cell indicates the probability of each treatment being ranked from first (high value) to last (low value) on progression-free survival (PFS), overall survival (OS), disease control rate (DCR), and grade 3 or higher adverse events (⩾3 AEs) in primary and sensitive analyses.

Supplementary Figure S5. Sensitive analysis of network meta-analysis on PFS, OS, DCR, and ⩾3 AEs by excluding studies with 100% of previously treated with targeted therapy.

A

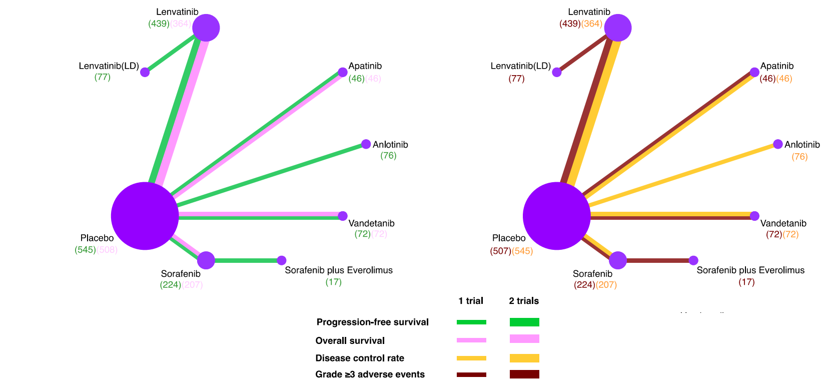

B

Progression-free survival (upper triangle) / **Overall survival** (lower triangle)

**Apatinib**	1.24 (0.54, 2.84)	1.34 (0.69, 2.59)	0.93 (0.37, 2.33)	0.68 (0.22, 2.07)	**0.44 (0.23, 0.85)**	**0.41 (0.20, 0.84)**	**0.26 (0.14, 0.48)**
	**Anlotinib**	1.08 (0.58, 2.01)	0.75 (0.31, 1.83)	0.55 (0.18, 1.63)	**0.36 (0.19, 0.66)**	**0.33 (0.17, 0.66)**	**0.21 (0.12, 0.37)**
0.63 (0.25, 1.59)		**Lenvatinib**	0.69 (0.37, 1.32)	0.51 (0.19, 1.34)	**0.33 (0.23, 0.48)**	**0.31 (0.20, 0.49)**	**0.19 (0.15, 0.25)**
			**Lenvatinib(LD)**	0.73 (0.23, 2.34)	**0.47 (0.23, 0.99)**	**0.44 (0.20, 0.97)**	**0.28 (0.14, 0.56)**
				**Sorafenib plus Everolimus**	0.65 (0.26, 1.60)	0.61 (0.22, 1.67)	**0.38 (0.15, 0.98)**
0.53 (0.21, 1.33)		0.84 (0.48, 1.47)			**Sorafenib**	0.94 (0.59, 1.48)	**0.59 (0.45, 0.77)**
0.51 (0.19, 1.33)		0.81 (0.44, 1.49)			0.96 (0.52, 1.78)	**Vandetanib**	**0.63 (0.43, 0.92)**
**0.42 (0.18, 0.97)**		**0.67 (0.45, 1.00)**			0.8 (0.54, 1.19)	0.83 (0.52, 1.33)	**Placebo**

C

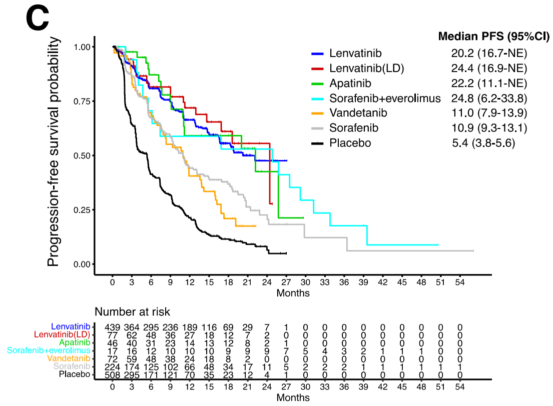

D

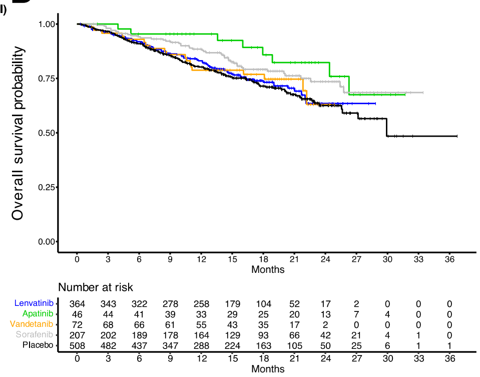

E

Grade 3 or higher adverse events (upper triangle) / **Disease control rate** (lower triangle)

**Apatinib**		0.62 (0.19, 2.08)	0.74 (0.20, 2.93)	4.42 (0.47, 41.10)	**4.40 (1.46, 14.41)**	**3.59 (1.01, 13.72)**	**18.35 (6.71, 55.94)**
1.57 (0.13, 19.3)	**Anlotinib**						
3.01 (0.68, 23.6)	1.92 (0.40, 15.99)	**Lenvatinib**	1.19 (0.62, 2.28)	7.17 (0.94, 53.49)	**7.12 (3.66, 14.23)**	**5.83 (2.29, 14.58)**	**29.66 (17.82, 51.93)**
			**Lenvatinib(LD)**	6.02 (0.70, 49.89)	**5.96 (2.35, 15.39)**	**4.88 (1.57, 15.01)**	**25.02 (10.78, 58.53)**
				**Sorafenib plus Everolimus**	0.99 (0.15, 6.82)	0.81 (0.10, 6.64)	**4.17 (0.59, 29.85)**
**8.37 (1.92, 65.76)**	**5.37 (1.11, 44.33)**	**2.78 (1.54, 5.04)**			**Sorafenib**	0.82 (0.34, 1.92)	**4.19 (2.78, 6.35)**
**10.24 (2.13, 85.56)**	**6.56 (1.22, 57.88)**	**3.40 (1.53, 7.54)**			1.22 (0.56, 2.64)	**Vandetanib**	**5.11 (2.45, 11.05)**
**18.32 (4.54, 140.26)**	**11.78 (2.58, 94.18)**	**6.12 (3.97, 9.56)**			2.20 (1.48, 3.28)	1.81 (0.93, 3.52)	**Placebo**

(A) Network diagrams of comparisons on progression-free survival (PFS), overall survival (OS), disease control rate (DCR), and grade 3 or higher adverse events (⩾3 AEs). (B) Pooled estimates of the network meta-analysis of PFS and OS. Data in each cell is Hazard ratio (HR) (95% CrIs) for the comparison of row-defining treatment versus column-defining treatment. HR less than 1 favors upper-row treatment. Significant results are highlighted in bold. (C) Reconstructed Kaplan-Meier curve of PFS for each treatment with median PFS. (D) Reconstructed Kaplan-Meier curve of OS for each treatment. (D) Pooled estimates of the network meta-analysis of DCR and (⩾3 AEs. Data in each cell is Odds ratio (OR) (95% CrIs) for the comparison of row-defining treatment versus column-defining treatment. OR greater than 1 favors upper-row treatment. Significant results are highlighted in bold.
